# Computational-aided drug design strategies for drug discovery and development against oral diseases

**DOI:** 10.3389/fphar.2025.1678652

**Published:** 2025-11-07

**Authors:** Tong Wu, Wentao Jiang

**Affiliations:** 1 School of BioSciences, Faculty of Science, University of Melbourne, Parkville, VIC, Australia; 2 School of Stomatology, Shandong Second Medical University, Weifang, China

**Keywords:** computer-aided drug design, oral diseases, peptide, small molecule, plant extract

## Abstract

Oral diseases, including dental caries, periodontitis, oral cancer, and mucosal infections, significantly impact overall health, underscoring the need for effective drug development. However, the discovery of novel oral drugs remains challenging due to complex disease mechanisms and limitations in traditional drug screening methods. Computer-aided drug design (CADD) has emerged as a powerful technology to accelerate drug discovery by improving efficiency and reducing costs. This review explores the application of CADD in the development of peptide-based drugs, small molecules, and plant extracts for oral diseases. It discusses CADD-associated antibacterial, anti-inflammatory, anticancer, and tissue regeneration therapies, highlighting available models, online tools, and successful case studies. Additionally, this review examines the intersection of CADD with natural product-based drug discovery, expanding therapeutic possibilities. While CADD enhances drug discovery, challenges such as mismatches in virtual screening and the need for experimental validation remain to be overcome. Despite these limitations, CADD is gaining traction in oral medicine, with the potential to revolutionize treatment strategies. This review aims to inspire further research and promote innovative therapeutic approaches to improve oral health and patient outcomes by summarizing recent advancements and emerging trends.

## 1 Introduction

The oral cavity serves not only as the starting point of the digestive system but also as a critical site for the manifestation of various diseases. The oral cavity has unique diseases, such as dental caries, periodontitis, pulpitis, and periapical periodontitis. Additionally, studies have shown a close relationship between oral health and overall health, as oral diseases can impact distant organs through mechanisms such as chronic inflammation, bacteremia, and immune system responses (Ferreira et al., 2015; Gupta et al., 2022). For example, they may increase the risk of cardiovascular diseases or be associated with the pathogenesis of Alzheimer’s disease. Furthermore, oral diseases affect mental health and social functions, potentially leading to anxiety, low self-esteem, and reduced quality of life (Gupta et al., 2022).

The design of oral drugs relies primarily on traditional small-molecule drug development, including empirical methods and chemical synthesis-based approaches. However, this conventional strategy often takes long screening cycles, high research and development costs, and low success rates, making large-scale clinical applications challenging (Humphrey et al., 2008). Additionally, research on oral disease drugs has lagged behind that in other medical fields because of the complexity of oral microenvironment and specificity of local drug delivery methods (Kamer et al., 2008). Thus, improving the efficiency of oral drug development remains a key challenge. In terms of pathology, dental caries is primarily driven by *Streptococcus mutans*, a cariogenic bacterium with strong acidogenic and biofilm-forming capabilities (Loesche, 1986; Bowen and Koo, 2011). whereas *Porphyromonas gingivalis* is a major pathogenic species in periodontitis (Coats et al., 2009). Oral inflammation and oral cancers are further linked to dysregulated host signaling pathways, including MAPK, NF-κB, and PI3K-Akt cascades (Liu Y. et al., 2024). These concise notes on etiology provide essential biological context for understanding how CADD-based strategies can be applied to oral disease therapeutics.

**FIGURE 1:**
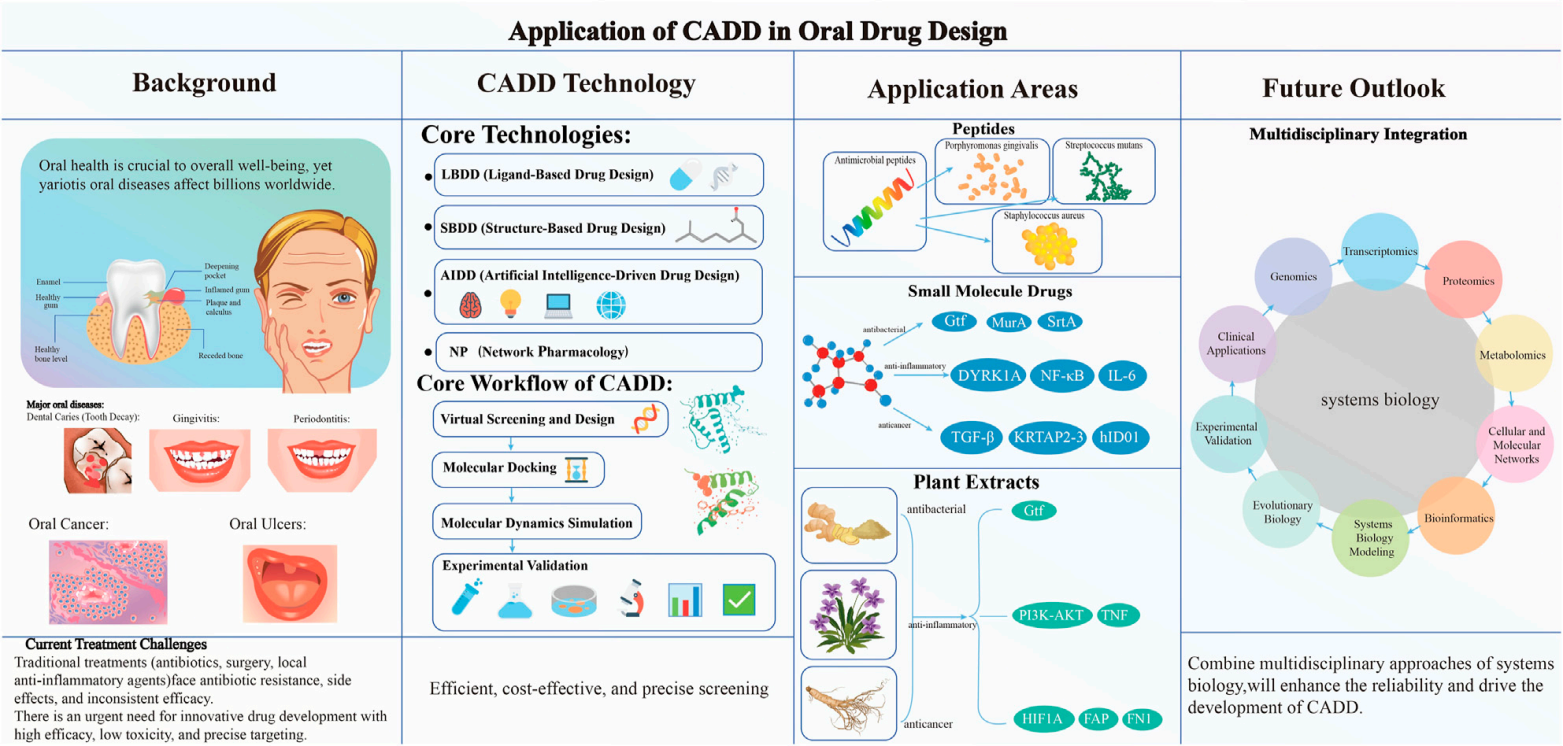
A schematic illustration of this review, which is intended to discuss CADD technologies and related drugs in oral disease treatment.

CADD uses computational methods such as molecular docking, molecular dynamics (MD) simulation, and virtual screening (VS) to efficiently predict drug‒target interactions, significantly reducing development time and improving success rates. Moreover, advancements in artificial intelligence (AI) and machine learning (ML) have further enhanced the predictive capabilities of CADD, increasing the precision of drug screening and optimization (Shoichet, 2004). Consequently, the application of CADD in the development of oral disease drugs holds excellent promise, accelerating the discovery of novel therapeutics and enhancing treatment efficacy. This review explores the application of CADD in the development of drugs for oral diseases, examining its potential, challenges and future advancements (Figure 1). Moreover, this review provides lists of easily accessible models useful for the drug development for oral diseases.

Nevertheless, despite these advances, current research efforts remain unevenly distributed, with most studies focusing on antibacterial strategies while neglecting anti-inflammatory, antitumor, and tissue regeneration approaches, highlighting the urgent need for a more critical and comprehensive evaluation of CADD applications in oral diseases.

## 2 What is CADD?

CADD technology utilizes computer techniques and computational methods to accelerate and optimize the drug development process (Lavecchia and Di Giovanni, 2013). It simulates the structure, function, and interactions of target molecules with ligands to screen, design, and optimize potential drug compounds. These approaches often integrate molecular docking, molecular dynamics simulations, pharmacophore modeling, and virtual screening, which allow researchers to explore binding conformations, calculate binding affinities, and evaluate molecular stability under near-physiological conditions (Meng et al., 2011). By narrowing down the number of experimental candidates, CADD not only reduces research costs and development cycles but also improves the precision of hit identification and lead optimization (Schneider, 2018). This article covers various CADD techniques, including ligand-based drug design (LBDD), structure-based drug design (SBDD), artificial intelligence-driven drug discovery (AIDD), and other related methods. LBDD guides drug optimization and novel drug design by studying the structure-activity relationships (SARs) of known ligands. Methods include quantitative structure-activity relationship (QSAR), which predicts the activity of new molecules on the basis of mathematical models that correlate chemical structures with biological activity (Yu and MacKerell, 2017). ML models, such as variational autoencoders (VAEs) and generative adversarial networks (GANs), are used to generate new compounds with desired properties (Sanchez-Lengeling and Aspuru-Guzik, 2018). SBDD leverages the three-dimensional structural information of macromolecular targets to identify key binding sites and interactions, designing drugs that can interfere with critical biological pathways (Anderson, 2003). Additionally, molecular dynamics can refine docking results by simulating atomic motions over time, while pharmacophore models provide generalized interaction patterns that facilitate the identification of novel scaffolds (Yang, 2010). Consensus or hybrid-based drug design combines multiple strategies, such as integrating LBDD and SBDD, to overcome the limitations of individual approaches. By leveraging complementary methods, it improves prediction accuracy and enhances the robustness of hit identification (Scior et al., 2012).

**FIGURE 2:**
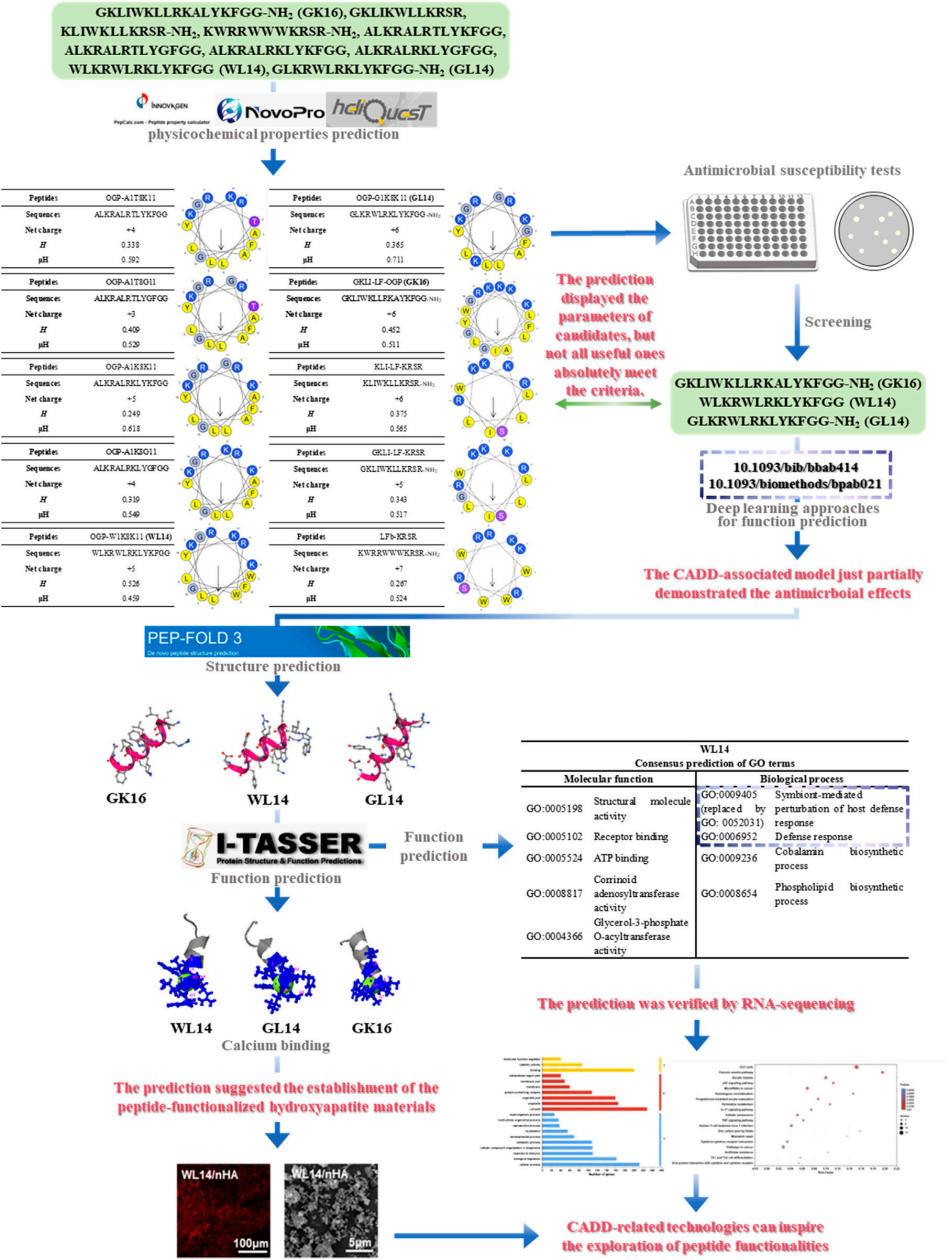
An instance for how CADD-associated online tools help improve the research. The physicochemical properties, structures and biological activities of a group of peptides were predicted with the online tools.

In this context, computer-aided drug discovery (CADD) serves as the broader computational framework, within which AI-driven drug discovery (AIDD) has emerged as an advanced subset that explicitly integrates artificial intelligence (AI) and machine learning (ML) into key steps such as candidate generation, ranking, and drug–target interaction prediction. Thus, AIDD represents the progression from traditional computational methods toward more intelligent and adaptive paradigms, embedded within the overarching CADD framework. Classical CADD techniques include molecular docking, which predicts the binding modes of small molecules to targets, and virtual screening (VS), which computationally filters large compound libraries to identify candidates with desired activity profiles (Yu and MacKerell, 2017). High-throughput virtual screening (HTVS) extends these approaches by combining docking, pharmacophore modeling, and free-energy calculations to enhance efficiency (Meng et al., 2011; Lionta et al., 2014). when AI/ML is incorporated to pre-filter compounds or re-rank docking results, HTVS exemplifies the application of AIDD within CADD. Additional pillars include molecular dynamics (MD) simulations and free-energy protocols for pose refinement, as well as ligand-based modeling approaches such as quantitative structure–activity relationship (QSAR) and pharmacophore analysis for activity inference. Network pharmacology (NP) further integrates systems-level biological data with CADD outputs to elucidate mechanisms, identify novel targets, and design multitarget drugs (Hopkins, 2008; Luo et al., 2020). When enhanced with AI—for tasks such as network construction, multi-omics integration, or mechanistic inference these pipelines likewise exemplify AIDD embedded within CADD (Zhou et al., 2019; Li and Zhang, 2013). As illustrated in Figure 2, although these computational models generate theoretical predictions, they often do not fully match experimental results. Similar discrepancies have been reported in other studies. In this study, 63 APRs were identified from the *S. mutans* proteome, and 54 peptides were synthesized, but only three (C9, C12, and C53) displayed significant antibacterial activity (Chen et al., 2024).This highlights a recurring gap across studies: while computational screening provides valuable hypotheses, many predicted hits remain theoretical, overly complex to validate, or even impossible to confirm experimentally. Taken together, this layered view (CADD → AIDD → technique-level tools) provides the conceptual basis for subsequent discussions of established oral applications (Section 2) and translational prospects (Section 3).

### 2.1 Widely used structure-prediction models for peptides/proteins

AlphaFold is a popular deep learning model developed by DeepMind to predict the three-dimensional structure of proteins, addressing the long-standing protein folding problem (Institute, EB, 2025). The model leverages amino acid sequences, evolutionary information, multiple sequence alignments (MSAs), coevolutionary data, template sequences, and structural information collaboratively through a multitrack attention mechanism, significantly improving prediction accuracy (Yu and MacKerell, 2017). The latest version, AlphaFold 3, has enhanced the prediction of protein interactions with other biomolecules, providing a new tool for studying complex biological processes and disease mechanisms (Booth, 2024). An analysis of the precise structure of PD-1 has optimized the Dostarlimab antibody used to treat endometrial cancer and other tumors (Canon et al., 2019). It has also aided in understanding KRAS conformational changes, improving the design of the KRAS G12C inhibitor Sotorasib (Canon et al., 2019). Additionally, AlphaFold has resolved the active site structures of EGFR mutations, enhancing the efficacy of the breast cancer drugs Erlotinib and Gefitinib (Zhang et al., 2006). In diabetes treatment, AlphaFold has revealed the three-dimensional structure of the GLP-1 receptor, optimizing the targeting of Semaglutide (Zhang et al., 2017).

RaptorX predicts residue-residue contact probabilities through MSA, enabling accurate modeling of the three-dimensional structures of proteins without homologous templates (Raptor, 2025). It can identify active sites and optimize drug design, such as improving multitarget kinase inhibitors (e.g., Lenvatinib) in cancer drug development to block tumor-related protein activity (Shaikh et al., 2021; Ramsay et al., 2018). DeepAccNet is used to assess and optimize protein structure quality, excelling in the quantitative analysis of local accuracy (Hiranuma et al., 2021). Improving protein structure prediction and facilitating the screening of candidate drugs such as remdesivir and GS-441524 plays key roles in the development of antiviral drugs for the treatment of COVID-19 (Zhuang and Ibrahim, 2021; Kim et al., 2021). ESMFold accelerates drug development with lower computational resources, aiding target identification and vaccine design in COVID-19 research. Its high prediction accuracy is advantageous for studying complex proteins such as protein kinases and farnesyltransferases (Herrington et al., 2024).

### 2.2 Easily accessible online models for peptides

Currently, in addition to large commercial models, numerous online small-scale model resources are available for researchers. As shown in Table 1, the online model resources for peptides/proteins are listed. These tools can conveniently assist in the research of peptide/protein drugs.

**TABLE 1:** Easy accessible online CADD-associated models for proteins or peptides.

Model	Corresponding molecule	Application	Technology	Website
AlphaFold (Laboratory EMB, 2025)	Proteins/Peptides	High-precision 3D protein structure prediction is suitable for complex proteins and long peptides and is widely applied in drug development and functional research	Utilizing deep learning and multiple sequence alignment (MSA)-derived evolutionary information, combined with neural networks and attention mechanisms to predict protein tertiary structures, achieving atomic-level accuracy by learning geometric relationships between residue pairs	https://alphafold.ebi.ac.uk/
RoseTTAFold (Laboratory B, 2025)	Proteins/Peptides	Lightweight and rapid protein structure prediction, suitable for well-defined functional domains and routine predictions	Employing multimodal networks (multichannel convolution and attention mechanisms) and sequence pairwise constraints to efficiently predict protein 3D structures, with the ability to process smaller datasets and generate diverse models	https://robetta.bakerlab.org/
I-TASSER (Zhang, 2024)	Proteins/Peptides	Homology modeling-based prediction integrates active site and functional annotations, making it suitable for function-oriented studies	Combining threading-based template alignment with fragment assembly methods, utilizing templates to guide protein folding, and employing low-resolution energy functions and MD simulations to refine structures	https://zhanggroup.org/I-TASSER/
Swiss-Model (Swiss-Model, 2025)	Proteins/Peptides	Online homology modeling tools are suitable for preliminary research and low-resolution modeling requirements	A web-based service where users upload sequences, and the tool automatically searches for templates, aligns sequences, and constructs 3D models using energy minimization to optimize the final structure	https://swissmodel.expasy.org/
RaptorX (Xu, 2025)	Proteins/Peptides	Suitable for structure prediction of proteins with low sequence similarity, supporting secondary structure, contact map, and solvent accessibility analysis	Utilizing deep learning models to predict residue contact maps and distance constraints, particularly effective for remote homology proteins	https://www.raptorx.com/
Quark (Zhang, 2025)	Proteins/Peptides	*De novo* protein structure modeling, suitable for template-free sequences and exploratory research	Employing a physics-based energy function and Monte Carlo sampling based on an *ab initio* approach, to simulate the protein folding process and generate tertiary structures	https://zhanggroup.org/QUARK/
ColabFold (Mirdita et al., 2022)	Proteins/Peptides	A simplified version of AlphaFold for structure prediction	An open-source adaptation of AlphaFold, optimized for speed and integrating MSA-based lightweight prediction workflows, enabling execution on Google Colab	https://colabfold.mmseqs.com/
Intfold (Mirdita et al., 2022)	Proteins/Peptides	Providing comprehensive predictions, including 3D structure, disordered regions, functional domains, and ligand-binding sites	Integrating multiple predictive algorithms, and leveraging MSA and energy scoring to enhance prediction accuracy	https://www.reading.ac.uk/bioinf/IntFOLD/
DeepFold (Lab Z, 2025a)	Proteins/Peptides	*De novo* 3D protein structure prediction, suitable for molecules with complex folding patterns	Utilizing deep learning frameworks to analyze amino acid sequence features and generate protein 3D structures	https://zhanggroup.org/DeepFold/
Phyre2 (Kelley et al., 2015)	Proteins/Peptides	Structure modeling for challenging-to-predict proteins	Employing template-driven prediction, and integrating Hidden Markov Models (HMM) to generate structural models for low-homology sequences	https://www.sbg.bio.ic.ac.uk/phyre2/html/page.cgi?id=inde
CABS-dock (University of Warsaw BG, 2025)	Protein-peptide complexes	Simulating protein‒ligand (e.g., small molecules, peptides) docking to predict binding modes	Using an elastic network model to simulate peptide docking on protein surfaces, and predicting flexible peptide binding sites and conformations	https://biocomp.chem.uw.edu.pl/CABSdock
GRAMM (Kansas Uo, 2025)	Protein‒protein complexes	Tool primarily used for molecular docking analysis, particularly in PPI, and identifying potential docking structures, aiding in protein interaction studies, drug design, and molecular biology research	Using a global search algorithm and fast Fourier transform (FFT), efficiently predicting protein‒protein docking conformations by assessing molecular surface complementarity	https://gramm.compbio.ku.edu/
PEP-FOLD3 (Lamiable et al., 2016)	Short peptides (≤25 residues)	Predicting the 3D structure of short peptides, applicable to peptide drug development and epitope research	Specialization in short peptide modeling using fragment-based MD simulations and generating peptide conformations for interactions with target proteins	https://bioserv.rpbs.univ-paris-diderot.fr/services/PEP-FOLD3/
ProtParam (Lamiable et al., 2016)	Peptides/Proteins	Analyzing physicochemical properties of proteins (e.g., molecular weight, isoelectric point, extinction coefficient)	Computing physical and chemical properties of protein sequences, including molecular weight, isoelectric point, and hydrophobicity, for rapid sequence characterization	https://web.expasy.org/protparam/
DynaMut2	Proteins/Peptides	Evaluating the impact of specific mutations on protein stability (^ΔΔ^G values) and predicting flexibility changes	Employing MD simulations and elastic network models to assess the structural and functional effects of mutations	https://biosig.lab.uq.edu.au/dynamut2/
DeepMSA (Zhang et al., 2020)	Proteins	Generating high-quality MSA, particularly for distantly related homologous proteins, aiding structural prediction, functional annotation, and evolutionary analysis	Integrating deep learning with sequence alignment tools (HHblits, JackHMMER, DeepCS) to enhance remote homology detection	https://seq2fun.dcmb.med.umich.edu//DeepMSA/
SPARKS-X (Lab S, 2024)	Protein/Peptides	Generating three-dimensional protein structures through template-driven modeling, particularly utilizing remote homology templates	Employing statistical potential and neural networks to predict protein contact maps, which are then integrated with geometric optimization to construct structural models	https://sparks-lab.org/server/sparks-x/
Psipred (Jones, 2025)	Proteins/peptides	Predicting α-helices, β-strands, and coil regions, and serving as an essential preliminary step for protein functional analysis and three-dimensional modeling	Utilizing a neural network-based approach to analyze sequence information, achieving high accuracy in predicting secondary structural elements	bioinf.cs.ucl.ac.uk/psipred
HHpred (Biology MPIfD, 2025)	Proteins	Identifying distant homology relationships among protein sequences to predict three-dimensional structures and functional properties and serving for protein family classification and function prediction	Employing HMMs for MSA and HMM-HMM comparison to enhance the accuracy of homology detection	https://toolkit.tuebingen.mpg.de/tools/hhpred
Modeller (Lab Z, 2025b)	Proteins/peptides	A comparative modeling tool used for structure prediction when template structures are available and applicable for modeling peptide fragments	Utilizing geometric constraints and optimization algorithms to generate structural models, supporting multitemplate modeling	https://salilab.org/modeller/
SwissSidechain (Sidechain, 2025a)	Side-chain modifications for protein engineering	Facilitating the study of nonnatural amino acid side chains in peptides and proteins and supporting molecular mechanics analysis and simulations	Provides structural files and MD parameters for nonnatural side chains	https://www.swisssidechain.ch/
RCSB PDB (Bank R PD, 2025)	Proteins	A model designed for accurate protein structural prediction and identification, playing a crucial role in studying PPI, functional characterization, and drug design and predicting potential therapeutic targets	Integrating deep learning techniques, combining convolutional neural networks (CNNs) and recurrent neural networks (RNNs) to analyze sequence data and generate precise structural models, and incorporating MD simulations and molecular docking techniques to refine protein‒target predictions for drug discovery	https://www.rcsb.org/
EVcouplings (Team, 2025)	Proteins	Predicting protein structures, PPI, and protein‒ligand interactions, and serving for identifying critical residues and functional sites	Employing coevolutionary analysis to reveal covariation relationships between residues, thereby inferring three-dimensional protein structures, based on aligning a large number of homologous protein sequences and identifying coevolving residue pairs, which are then applied in structure prediction and functional analysis	https://evcouplings.org/
Protter (Omasits et al., 2014)	Proteins and transmembrane peptides	Providing a graphical representation of protein topology, including transmembrane regions and signal peptides, aiding in protein function and localization studies	Offering a two-dimensional visualization of transmembrane regions and functional domains, facilitating the interpretation of experimental data	https://wlab.ethz.ch/protter/start/
DeepTracer (University of Washington, 2025)	Proteins (particularly macromolecular complexes), nucleic acids (potentially included in complexes)	Predicting the structure of multichain protein complexes from cryo-electron microscopy (cryo-EM) density maps	Deep learning-based approach enabling rapid full-atomic structure prediction, particularly suitable for viral protein research	https://deeptracer.uw.edu/home
ProteinPredict (PredictProtein, 2025)	Proteins	Predicting protein secondary structures, solvent accessibility, transmembrane regions, intrinsically disordered regions, functional annotations (e.g., GO terms), and binding sites	Integrating evolutionary information, MSA, neural network algorithms, and deep learning techniques (e.g., MMseqs2 for computational acceleration)	https://predictprotein.org/
CASP Tools (Center, 2025)	Proteins/peptides	Evaluating and benchmarking the performance of global protein structure prediction methods	Comparing predicted structures with experimentally determined reference structures (obtained via X-ray crystallography, NMR spectroscopy, or cryo-EM)	https://predictioncenter.org/
Moe (Group, 2025)	Proteins/peptides	Providing molecular docking, MD simulations, drug molecule optimization, and protein engineering design	Offering comprehensive molecular modeling and simulation functionalities, including force field optimization, energy minimization, and MD simulations	https://www.chemcomp.com/en/index.htm
GRAMM (Group UoKCB, 2025)	Protein‒protein complexes	Serving for molecular docking analysis, specifically for PPI, and identifying potential docking conformations through global search	Utilizing global search algorithms and FFT to efficiently predict protein‒protein docking conformations, based on molecular surface matching to infer interaction patterns	https://gramm.compbio.ku.edu/

This review demonstrates how online tools optimize peptide research using a published study as an example (Figure 2) (Xie et al., 2025). In that study, 10 peptides were designed and analyzed via computational tools such as PepCalc, Heliquest, and NovoPro. Helical wheel diagrams revealed that GKLIKWLLKRSR, KLIWKLLKRSR-NH2, and KWRRWWWKRSR-NH2 lacked the amphipathic characteristics typical of antimicrobial peptides (AMPs), which was confirmed by antimicrobial susceptibility tests (Xie et al., 2025). WL14 and GL14 displayed potent antimicrobial activities, although they possessed abnormally predicted H and μH values. Interestingly, apart from GK16, peptides meeting the predicted criteria failed to show antimicrobial properties, highlighting discrepancies between theoretical predictions and experimental outcomes. Further analysis using the models of W. Tang et al. failed to predict the antimicrobial properties of WL14, GL14, and GK16 (Tang et al., 2022). In contrast, Gronning’s model revealed antibacterial but not antifungal activity (Grønning et al., 2021). Additional computational tools, such as PEP-FOLD and I-TASSER, provided deeper insights into the ability of WL14 to bind calcium ions and regulate host immune defense. These predictions were experimentally validated and helped develop a functionalized biomaterial with potent *in vivo* activity.

Overall, this study underscores the potential of CADD in peptide research while acknowledging its inherent limitations. While CADD provides a powerful framework for initial screening, it primarily relies on predictive models that are often inadequate for capturing the complexity of proteins and peptides with intricate three-dimensional conformations. At present, most algorithms are restricted to predictions of linear peptide sequences and lack the capacity to accurately model structurally complex or conformationally flexible peptides. Moreover, the predictive performance is constrained by parameter settings, limited training datasets, and oversimplified assumptions, which may lead to discrepancies between *in silico* predictions and actual biological activities. Consequently, some effective peptides may be overlooked, while others predicted to be active may ultimately prove ineffective when validated experimentally.

### 2.3 Widely used structure-prediction models for small molecules

AutoDock Vina is a molecular docking tool designed to predict the binding modes and affinities of small molecules with target proteins. It is widely utilized in VS and lead compound optimization. For example, in the screening of COVID-19 inhibitors, potential inhibitors such as Remdesivir metabolites have been identified (Trott and Olson, 2010). The Schrödinger Suite is an advanced molecular simulation and drug design platform for molecular modeling, VS, and MD studies (Schrödinger, 2025).

### 2.4 Easily accessible online models for small molecules

This section also covers commonly used online predictive models for small molecules (Table 2). These models integrate big data and ML techniques, significantly increasing the efficiency of candidate compound screening and optimization, thereby providing robust support for drug discovery and development. In NP research, online predictive models are widely used for molecular property prediction, activity assessment, and VS, significantly improving the efficiency of candidate compound selection and optimization and thus providing strong support for drug discovery and development. For example, a study on the potential therapeutic mechanism of *Panax ginseng* for periodontitis utilized SwissTargetPrediction to predict the targets of its active compounds (Sun et al., 2024). This study revealed its potential role in immune regulation and anti-inflammatory effects by integrating NP and molecular docking analyses. Another study on *Pueraria flowers* and *Hovenia dulcis* in the treatment of alcohol-induced liver injury combined SwissTargetPrediction target prediction with protein–protein interaction (PPI) network analysis and GO and KEGG pathway enrichment analysis to investigate their therapeutic potential (Wang et al., 2020). These studies highlight the crucial role of online predictive tools in NP research by facilitating the mechanistic exploration of traditional Chinese medicine (TCM) and providing valuable data support for modern drug research.

**TABLE 2:** Easy accessible online CADD-associated models for small molecules.

Model	Corresponding molecule	Application	Technology	Website
SwissADME (Daina et al., 2025)	Small molecule compounds	Predicting drug-like properties of small molecules, including drug-likeness and ADME (Absorption, Distribution, Metabolism, and Excretion) properties, and aiding drug screening and optimization	Utilizing rule-based computations (e.g., Lipinski’s rule), molecular descriptors, and predictive models	http://www.swissadme.ch/
MolSoft Drug-Likeness (LLC, 2025)	Small molecule compounds	Assessing drug-likeness, toxicity, and activity of molecules, and facilitating early-stage drug candidate screening	Employing molecular descriptors (e.g., LogP, TPSA) and drug chemistry evaluation rules	https://molsoft.com
DeepChem (DeepChem, 2025)	Small molecules, proteins, and complexes	Providing a machine learning framework for molecular modeling, drug discovery, and chemical property prediction	Leveraging deep learning techniques, supporting neural network model construction, and providing chemical datasets (e.g., SMILES, graphical representations)	https://deepchem.io/
PatchDock (Project OB, 2025)	Protein‒protein and protein-small molecule docking	Facilitating docking of proteins and small molecules, particularly suitable for shape-complementary docking	Utilizing a shape-matching algorithm, which generates docking candidates through geometric analysis of surface features and optimizes results by scoring functions	https://bioinfo3d.cs.tau.ac.il/PatchDock/
ProTox-II (Banerjee et al., 2018)	Small molecule compounds	Predicting compound toxicity, including acute toxicity (e.g., LD50 values), toxicity categories (e.g., low or high toxicity), and specific toxicity targets (e.g., hepatotoxicity, carcinogenicity)	Providing machine learning-based, integrates multiple toxicity databases (e.g., ChEMBL, ZINC), supporting chemical structure input, and offering a user-friendly interface for toxicity analysis	https://tox.charite.de/protox3/#
ADMETlab 2.0 (Xiong et al., 2021)	Small molecule compounds	Providing comprehensive predictions of ADMET properties for drug design and optimization, and improving the success rate of candidate drugs	Providing deep learning-based, integrates multiple ADMET property databases, supporting drug-likeness rule filtering (e.g., Lipinski’s rule), and offering optimization recommendations with multidimensional analysis	https://admetmesh.scbdd.com/
ChemBioServer (Founda and tion, 2025)	Small molecule compounds	Managing and screening chemical compound libraries, filtering out highly toxic compounds and optimizing drug candidate selection workflows	Supporting multiple filtering rules (e.g., Lipinski’s rule, toxicity filters), and enabling rapid screening of compounds that meet drug-likeness and safety standards, suitable for HTVS	https://chembioserver.vi-seem.eu/
pkCSM (Pires, 2015)	Small molecule compounds	Predicting drug pharmacokinetics and ADMET properties, including absorption, permeability, volume of distribution, and protein binding affinity, and aiding the assessment of drug safety and efficacy	Employing a graph-based machine learning model using molecular SMILES input with a user-friendly interface	https://biosig.lab.uq.edu.au/pkcsm/
eMolTox (Xund. Moltox, 2025)	Small molecule compounds	Predicting molecular toxicity (e.g., mutagenicity, carcinogenicity, environmental toxicity) and pharmacological properties, and enhancing compound drug design	Combining QSAR models and statistical approaches, analyzing molecular structural properties, and supporting interactive visualization of results for scientific interpretation	https://xundrug.cn/moltox
OpenBabel (Project OB, 2025)	Small molecule compounds	A cheminformatics toolkit for molecular format conversion, molecular modeling, and chemical database querying	Supporting multiple molecular format conversions as an open-source and cross-platform, and providing both command-line and Python interfaces	https://openbabel.org/
AlphaFold-Multimer (Institute, EB, 2025)	Protein and small molecule complexes	Modeling the three-dimensional structures of multimeric complexes, and predicting PPI and protein-small molecule interactions	Deep learning-based structural prediction, utilizing the AlphaFold algorithm with a focus on multimeric systems	https://alphafold.ebi.ac.uk/
Schrödinger (Schrödinger, 2025)	Small molecules and protein targets	A comprehensive molecular modeling and simulation platform for drug discovery, including docking, free energy calculations, and MD.	Integrating quantum mechanics, MD, docking algorithms, and cheminformatics tools	https://www.schrodinger.com/
Rosetta (RosettaCommons, 2025)	Chemical molecules, small molecules, carbohydrate molecules	A protein design and modeling tool capable of predicting protein-small molecule interactions, protein folding, and docking	Getting used in protein engineering, drug docking studies, and biomolecular design	https://rosettacommons.org/
LigandScout (Inte, 2025)	Small molecules and target interactions	Ligand-based pharmacophore modeling, used for VS and initial screening in drug discovery	Supporting VS, hit identification based on pharmacophores, and prioritization of lead compounds	https://www.inteligand.com/ligandscout/

Specific tools possess broad applicability, can predict peptide-related properties, and are suitable for small molecule analysis, exhibiting significant functional advantages for both research (Table 3).

**TABLE 3:** Easy accessible online CADD-associated models for proteins and small molecules.

Model	Corresponding molecules	Applications	Technology	Website
AlphaFold (Institute, EB, 2025)	Proteins, peptides Drug-like molecules	Used for protein structure prediction, inferring 3D structures from amino acid sequences. It is widely used in drug discovery, structural biology, and disease research. In small-molecule drug discovery, AlphaFold aids in SBDD, VS, and drug optimization by predicting target protein structures and interactions	A deep learning-based protein structure prediction tool using evolutionary information and MSA with a Transformer-based architecture. In small-molecule drug discovery, AlphaFold aids in target protein modeling, SBDD, and optimizing PPI.	https://alphafold.ebi.ac.uk/
Rosetta (RosettaCommons. Rosetta, 2025)	Peptides and small molecules	Rosetta is a computational toolkit for protein structure prediction, design, ligand docking, and MD, with applications in protein folding, enzyme design, and antibody design. It also optimizes ligand docking and binding affinity prediction, making it useful for protein engineering and drug discovery	Rosetta is an energy-function-based tool for protein structure prediction, enzyme engineering, and molecular docking, supporting small-molecule interactions and drug design. It enables flexible docking, ligand optimization, and SBDD; with user-defined constraints and parallel computing, Rosetta integrates experimental data for accurate modeling, making it a powerful tool for biomolecular design and therapeutic development	https://rosettacommons.org/
SwissSidechain (Sidechain, 2025b)	Small molecules and peptides	Serving for chemical modifications and side chain design, facilitating molecular optimization and synthetic feasibility assessment	It provides chemical fragment generation and optimization functionalities, supporting side-chain design and synthetic feasibility analysis, making it particularly suitable for medicinal chemistry research	https://www.swisssidechain.ch/
Molecular docking servers (e.g., SwissDock) (Sio, 2025)	Small molecules and peptides	Providing web-based molecular docking services eliminates the need for complex software installation and serves to predict binding modes between small molecules and target proteins rapidly	An online molecular docking tool based on AutoDock and similar algorithms, offering rapid binding mode prediction and affinity estimation without requiring intricate configurations	https://www.swissdock.ch/

## 3 The application of CADD in oral diseases

By employing CADD models in combination with database-based online tools, it is possible to accurately predict the conformations of proteins, peptides, and small molecules, thereby providing crucial technical support for novel drug screening. Building on this foundation, the present review systematically analyzes the potential value of these technologies and their research progress in drug development for oral diseases. In particular, we focus on the applications of CADD that have already been established in the oral field. Numerous CADD models have been applied to the design and optimization of diverse molecular drugs, including peptides, small molecules, and other bioactive compounds. These agents have been investigated for their antimicrobial, anti-inflammatory, and antitumor activities, targeting diseases such as dental caries, periodontitis, and oral squamous cell carcinoma. Accordingly, this review provides a systematic summary of CADD-based drugs that have been applied in the context of oral diseases.

### 3.1 Peptides

#### 3.1.1 Antimicrobial peptides

A wide range of oral diseases are closely associated with bacterial infections. Dental caries is a chronic, cumulative bacterial infectious disease, with *S. mutans* being the primary cariogenic pathogen. This bacterium exhibits acid production, acid tolerance, and biofilm-forming capabilities, making the development of antimicrobial compounds that target *S. mutans* a prominent research focus (Loesche, 1986; Bowen and Koo, 2011). In addition to dental caries, periodontitis is another highly prevalent chronic inflammatory oral disease and is a leading cause of tooth loss in adults. *Porphyromonas gingivalis* is recognized as the principal pathogenic bacterium in periodontitis and is characterized by its ability to invade host tissues, disrupt periodontal support structures, and evade immune responses (Hajishengallis, 2015). Among the various antimicrobial agents, AMPs have emerged as a research focus because of their broad-spectrum antimicrobial activity, low propensity for inducing resistance, and immunomodulatory functions (Wang et al., 2016).

Dongru Chen et al. utilized CADD technology to screen 14 hexapeptides from the sequence of the surface adhesion protein C123 of *S. mutans* and successfully synthesized 13 for experimental validation (Chen D. et al., 2021). They employed ZipperDB to evaluate whether each six-amino acid sequence could form amyloid fibrils. Tango was also used to predict the β-sheet propensity of peptides, assessing their potential for amyloid fibril formation. Moreover, Waltz was applied to evaluate amyloidogenicity, particularly for short peptide sequences. Five hexapeptides (P1, P3, P6, P7, and P13) were confirmed to have amyloid-forming capabilities. The experimental results demonstrated that these hexapeptides could bind to the cell wall components of *S. mutans*, thereby triggering amyloid fibril aggregation and subsequently inhibiting biofilm formation (Chen D. et al., 2021). Furthermore, these hexapeptides exhibit broad-spectrum antibiofilm activity against other gram-positive bacteria, such as *Streptococcus sanguinis*, gram-negative bacteria, such as *Escherichia coli*, and fungi, such as *Candida albicans* (Yang L. et al., 2023).

Chen Yucong et al. utilized the TANGO algorithm to screen the proteome of *S. mutans* and identified 63 seven-amino-acid-long aggregation-prone regions (APRs). These APRs were further compared via BLASTp to ensure that they matched multiple proteins within the *S. mutans* proteome (Chen et al., 2024). Among them, C9 and C12 exhibited significant antibacterial activity. Their mechanism of action includes inserting into the bacterial membrane, causing membrane disruption and increased permeability, as well as inducing intracellular protein aggregation, thereby accelerating bacterial cell death (Chen et al., 2024).

AMPs have been rarely explored for their efficacy against periodontal pathogens, particularly *P. gingivalis*, which may be associated with their secretion of proteases. By degrading protein-based therapeutics, these enzymes serve as key factors in the destruction of periodontal tissues and play crucial roles in conferring resistance to AMPs (Coats et al., 2009). Moreover, CADD helps screen for adequate AMPs that target *P. gingivalis*, suggesting the value of CADD in antimicrobial therapy.

Zarin Taj conducted a study to identify and optimize an AMP from *Lactobacillus* sp. via *in silico* approaches. They initially extracted 67 peptide sequences from multiple databases, including DRAMP (http://dramp.cpu-bioinfor.org/), dbAMP (https://awi.cuhk.edu.cn/dbAMP/), and DBAASP (https://dbaasp.org/home) (Taj and Chattopadhyay, 2024). These peptides were screened on the basis of their toxicity, bioactivity, and antibiofilm properties, leading to the selection of 12 candidate peptides. Among the selected peptides, plpl_18 was identified as the most promising AMP for targeting the periodontal pathogens, *P. gingivalis* and *Fusobacterium nucleatum*. Structural modeling and molecular docking studies confirmed its strong binding affinity for the virulence proteins RagB and Fap2, which play crucial roles in colonization and biofilm formation. MD simulations further validated the stability and specificity of p*lpl_18* in binding to these targets, suggesting its potential to inhibit key bacterial functions and reduce pathogenicity. Finally, wet laboratory experiments supported the *in silico* findings, highlighting the therapeutic potential of p*lpl_18* as an effective antimicrobial agent against periodontal pathogens (Taj and Chattopadhyay, 2024).

In addition to *P. gingivalis* and *S. mutans*, other clinically relevant pathogens, such as *Staphylococcus aureus*, also contribute to oral diseases, including oral mucositis and osteomyelitis (Peters et al., 2012; Jakubovics and Kolenbrander, 2010). A Protegrin 1-derived peptide, currently in a phase III clinical trial for oral mucositis, exemplifies the application of CADD in antimicrobial peptide optimization. Using QSAR models to predict antimicrobial activity, and ML algorithms such as support vector machines (SVM) and random forest (RF) to refine sequence selectivity and stability, these peptides achieve enhanced efficacy with reduced toxicity. Further improvements are achieved through genetic algorithms, *de novo* design, and pattern insertion strategies, which optimize antimicrobial potency and membrane-disrupting ability. Similarly, PAC-113 has been optimized with CADD tools to increase activity and stability while minimizing cytotoxicity. Molecular dynamics (MD) simulations validate the interactions of these peptides with bacterial membranes, ensuring both efficacy and safety. Collectively, these computational techniques have accelerated the development of novel AMPs, offering promising candidates for clinical applications (Cardoso et al., 2019).

Peptide drug development has advanced considerably over the past decade, driven by innovations in production, modification, and analytical technologies. Both chemical and biological methods, together with novel design and delivery strategies, have helped to address the inherent limitations of peptides and sustain progress in this field (Wang et al., 2022).In the context of oral diseases, computer-aided drug design (CADD) has facilitated the exploration of antimicrobial peptides (AMPs), particularly in infectious conditions. However, their application to oral inflammation and oral cancer remains minimal, leaving significant opportunities for further investigation. These peptides not only hold promise for modulating the oral inflammatory microenvironment and suppressing pathogenic infections, but also show potential as novel therapeutic strategies for oral malignancies. Despite this promise, no studies to date have reported the use of CADD-based peptides in oral inflammation, oral cancer, or other oral diseases, underscoring the urgent need for systematic research in this area.

### 3.2 Small molecules

In addition to AMPs, small-molecule compounds have emerged as another research focus. These compounds offer several advantages, including high structural diversity, ease of chemical modification, enhanced stability, and relatively low production costs. These properties underscore their significant potential in the development of anti-infective therapeutics. Unlike CADD-designed peptides lacking anti-inflammation and anticancer studies, CADD-based small molecules have demonstrated research potential not only in antimicrobial applications but also in anti-inflammatory and anticancer applications (Yang et al., 2018). For example, small molecule compounds can be designed to exert specific anti-inflammatory effects or to target the tumor microenvironment for antitumor efficacy (Zhong et al., 2020).

#### 3.2.1 Small molecules against oral pathogens

In the study of dental caries, antibacterial small molecules targeting *S. mutans* represent a significant direction in CADD-driven drug development (Liu P. et al., 2024; Hwang et al., 2017). Chen et al. utilized CADD for structure-based virtual screening (SBVS) to identify potential inhibitors targeting the C3 fragment of *S. mutans* from approximately 220,000 small molecules in the Specs database. Using molecular docking in MOE software, they calculated binding energies and applied Lipinski’s rule to filter the top 99 compounds with the highest binding affinities (Chen Y. et al., 2021). D25 exhibited strong inhibitory effects on *S. mutans* biofilm formation with minimal effects on commensal bacteria, such as *Streptococcus gordonii* and *S. sanguinis*, demonstrating good selectivity (Chen Y. et al., 2021). Further investigations revealed that D25 interferes with the interaction between the C3 fragment and A3VP1, leading to the formation of amorphous aggregates, disrupting the structural integrity of amyloid fibrils and ultimately destabilizing the biofilm. Transmission electron microscopy (TEM) revealed that D25 treatment made the amyloid fibrils surrounding *S. mutans* cells sparse and structurally abnormal. Additionally, srtA and pacR gene expression levels were significantly upregulated after D25 treatment, suggesting that D25 influences amyloid fibril formation through genetic regulation (Chen Y. et al., 2021).

Kanumuru et al. investigated the inhibitory effects of imidazole quinoline derivatives on Gingipain R, a major virulence factor of *P. gingivalis*. This study employed molecular docking to evaluate the binding affinity of these compounds and utilized SwissADME and ProTox II to predict their pharmacokinetic properties and toxicity risks (Reddy et al., 2023). Through AutoDock Vina, protein-ligand molecular docking was conducted to determine the binding modes of the imidazole quinoline derivatives 1-6. The results showed that compounds 2, 3, and 6 exhibited strong binding affinities, forming stable hydrogen bonds and hydrophobic interactions with the target protein Gingipain R. Additionally, SwissADME predictions confirmed that all the compounds adhered to Lipinski’s rule of five, indicating good oral drug development potential and demonstrating no blood-brain barrier permeability with a lower likelihood of central nervous system side effects. ProTox II toxicity assessment revealed no cytotoxicity, although some compounds exhibited hepatotoxicity or immunotoxicity.

Paul P. et al. aimed to identify small-molecule compounds capable of binding to the bacterial enzyme Pth1 (peptidyl-tRNA hydrolase) as identified through molecular docking simulations. Pth1 is crucial for bacterial survival but is nonessential in human cells, making it a promising target for oral antibacterial drug development (Reddy et al., 2023). Using virtual molecular docking screening, researchers have utilized existing crystal structure data for Pth1 and Pth2 to calculate the binding energies of various antibiotic molecules with these enzymes and rank the results accordingly. Some of the screened compounds, such as Cefixime and Cefoperazone, demonstrated broad-spectrum inhibitory potential across multiple Pth1 enzymes. Others exhibited narrow-spectrum inhibition, with selectivity toward specific bacterial Pth1 enzymes. For example, doxycycline was found to be selective for *Acinetobacter baumannii* Pth1. Moreover, most small molecules exhibited a significant ability to differentiate between Pth1 and Pth2, suggesting a reduced likelihood of off-target effects on human cellular enzymes. Molecular docking analysis revealed that different bacterial species exhibited subtle structural variations in their Pth1 enzymes, allowing small molecules to bind to these differences selectively. Furthermore, the core structures found in many antibiotics, such as the β-lactam ring in cephalosporins, may mimic the natural substrate of Pth1, thereby increasing its binding affinity. The development of Pth1-targeting drugs holds significant potential for the treatment of oral diseases. Oral infections such as gingivitis and periodontitis frequently involve Gram-positive and Gram-negative bacteria. Pth1 inhibitors could provide targeted antibacterial therapy with minimal impact on human cells (Reddy et al., 2023). Additionally, these compounds can be designed to exhibit either broad-spectrum or narrow-spectrum inhibitory activity, depending on therapeutic needs, thereby increasing treatment efficacy while mitigating antibiotic resistance. Furthermore, combining these novel inhibitors with existing treatments may prolong the effectiveness of current therapies by delaying the emergence of resistance.

Antimicrobial small molecules are a hot topic in CADD for oral applications. In addition to those mentioned above, many other oral CADD antimicrobial small molecules are listed in Table 4.

**TABLE 4:** Small molecules that can be used for oral diseases studied with CADD-associated technology.

Drug name	CADD-associated technology	Main experimental content
Antibacterial drugs		
2-(4-methoxyphenyl)-N-(3--1,4-dihydro-2-quinoxalinylidene)ethanamine (Ren et al., 2015)	Molecular docking screening was conducted using UCSF DOCK software to identify target molecules from a compound library	SBVS was conducted to identify small molecule inhibitors targeting Gtf of *S.mutans*, inhibiting biofilm formation and virulence. The efficacy was validated through *in vitro* assays and rat models, demonstrating reduced EPS synthesis and caries development
Astilbin (Wang et al., 2019)	Molecular docking and MD simulations were used to study Astilbin’s inhibition of SrtA. Docking predicts binding, while simulations assess complex stability. Binding free energy analysis (AmberTools 15) identifies Arg213, Leu111, and Leu116 as key interaction sites	Astilbin inhibits Sortase A (IC50 = 7.5 μg/mL) by targeting critical residues like Arg213, as shown by molecular docking and simulations. It reduces *S.mutans* biofilm formation by up to 70% without affecting growth, minimizing antibiotic resistance risks. By disrupting adhesion protein anchoring, astilbin helps prevent plaque and dental caries
ZI-187, ZI-939, ZI-906 (Rivera-Quiroga et al., 2020)	VS, molecular docking simulations, and MD simulations were employed to screen potential molecules targeting cariogenic bacteria	Three small molecules (ZI-187, ZI-939, and ZI-906) were identified via VS, demonstrating potent inhibition of *S.mutans* adhesion protein Ag I/II, with inhibition rates exceeding 90% and no observed toxicity. MD simulations and microscopic analyses validated their stable binding and significant antibacterial efficacy
2A4 (Liu et al., 2011)	SBVS was used to identify compounds that inhibit *S. mutans* biofilm formation. UCSF DOCK 6.4 was employed to screen 150,000 compounds from the ZINC database against the *GtfC* protein (PDB ID: 3AIC), followed by protein‒ligand interaction analysis using Chimera and LigPlot+	Compound 2A4 specifically inhibited *S.mutans* biofilm formation (IC50 = 0.94 µM), reducing biofilm thickness and adhesive molecule production without affecting commensal bacteria. It significantly altered the gene expression profile associated with *S.mutans*
Kurarinone (CHEMBL243796) (Salmanli et al., 2021)	Molecular docking and MD simulations were combined to analyze the binding mechanism of the compound with its target.	CADD-based screening identified SrtA inhibitors from a library of 178 compounds, yielding 163 suitable molecules, with six exhibiting high affinity for SrtA. Kurarinone emerged as the most potent candidate, forming stable interactions with key residues Arg213 and Cys205
ALS-31-9 (Cerchia et al., 2022)	LBVS and SBVS strategies were employed to optimize and identify compounds with enhanced inhibitory activity	For *S.mutans* superoxide dismutase (SmSOD), ALS-31 exhibited an IC50 of 159 µM. The mechanism of action involved disrupting critical residues (H166 and E165) and dimeric structure to inhibit enzyme activity. The optimized derivative ALS-31-9 demonstrated enhanced inhibitory efficiency with IC50 of 64 µM
6-Deoxy sucrose and Trichloro-galactosucrose (Alhobeira et al., 2021)	A quantum mechanics/molecular mechanics (QM/MM) approach, combined with flexible docking and ADMET analysis, was employed to optimize the inhibitor’s conformation and binding properties	Inhibitors targeting GtfC active sites (PDB ID: 3AIC) were identified using LigScore, with D-Acarbose as reference. Several inhibitors interact with key amino acids (Asp588, Trp517, Asn481), supported by ADMET predictions
LCG-N25 (Lyu et al., 2021)	Lead compound optimization and molecular design and synthesis were employed. Based on napabucasin (NAP), a novel small molecule LCG-N25 was designed and synthesized through key intermediate preparation and aminolysis. Using SBDD, molecular modifications enhanced antibacterial activity and safety, optimizing its anti-biofilm potential	LCG-N25 demonstrates excellent antibacterial activity and biofilm inhibition against oral streptococci with low toxicity and low resistance potential, making it a promising anti-caries agent
G43 (Zhang et al., 2017; Nijampatnam et al., 2021)	VS, SAR analysis, and multiple *in vitro* and *in vivo* experiments were conducted to validate the activity of the selected compounds	Small molecules such as G43 and IIIF1 target *S.mutans* Gtfs, selectively inhibiting biofilm formation, reducing caries scores, and exhibiting no toxicity or bactericidal effects on commensal bacteria
ZINC19924906 (ZI-906), ZINC95098840, ZINC99230413 (Luo et al., 2017)	A HTVS approach, coupled with MD simulations and ADMET evaluation, was employed to identify and validate potential inhibitors	Potential inhibitors targeting *S.mutans* SrtA were identified via virtual screening, MD, MM/PBSA binding energy calculations, and ADMET analysis. These inhibitors interact with key residues (Cys205 and Arg213), blocking SrtA activity and biofilm formation
Hydroxychalcone compound (Compound 9) (Nijampatnam et al., 2016)	Compounds were screened from a chemical library using molecular docking and multiple experimental techniques to analyze their effects	Compound 9 effectively inhibits Gtfs activity, blocking biofilm formation, and exhibits high selectivity and low toxicity *in vitro* and *in vivo* experiments
Imidazole Quinolines (Reddy et al., 2023)	Molecular docking analysis, SwissADME, and ProTox II tools were used to screen and evaluate the pharmacokinetics and toxicity of the compounds	Molecular docking analysis revealed imidazole quinolines exhibit superior binding affinity to Gingipain R of *P. gingivalis* protein compared to clinical drugs. Pharmacokinetics and toxicity assessments indicate that compounds 2, 3, and 6 are promising inhibitors
Five Oxazoles (oxazole) (Manikandan et al., 2023)	The study optimized compound structures using ChemOffice and performed pharmacokinetic analysis using AutoDock Vina and SwissADME.	Molecular docking analysis targeting *P. gingivalis* HmuY protein identified Compound 2 as the most potent inhibitor with stable binding. Some compounds require dose and structural optimization to reduce toxicity
Protocatechuic acid (Singh et al., 2024)	A HTVS strategy (Libdock), followed by molecular docking (CDOCKER) and MD simulation, was applied to identify potential inhibitors	Protocatechuic acid, identified from the NPACT database, binds FimA protein of *P. gingivalis*, effectively inhibiting its virulence associated with OSCC, with a binding energy of −61.65 kcal/mol and excellent stability
ZLS-31 (Cerchia et al., 2022)	A LBVS and SBVS strategy was implemented to optimize the activity of the selected compounds	ALS-31, identified through VS, effectively dissociates SmSOD dimer, inhibiting its activity while exhibiting low cytotoxicity. The derivative ALS-31-9 showed a 2.5-fold improvement in IC50
Anti-inflammatory drugs		
Isoetharine (Yadalam et al., 2022)	A systems biology approach, combined with PPI network analysis, molecular docking, and MD simulation, was used to identify key target compounds	Isoetharine binds DYRK1A with −4.70 kcal/mol binding energy, demonstrating stable interaction, suggesting potential use in periodontal regeneration
Anti-tumor drugs		
IHNOCS (Agar et al., 2024)	Molecular docking and MD simulation analyses were conducted to assess the stability of drug-target binding	Molecular docking and MD simulations confirm IHNOCS forms a TGF-β-KRTAP2-3 tri-complex, inhibiting KRTAP2-3 and modulating TGF-β to reduce oral cancer spread. IHNOCS binds KRTAP2-3 (−11.2 kcal/mol), suppressing migration, and modulates TGF-β via reversible hydrogen bonding (1.72 Å) to control proliferation
Cucumis maderaspatanus (Rao et al., 2024)	Gas chromatography‒mass spectrometry (GC‒MS) analysis and MD simulation were utilized to screen potential anticancer compounds	Active compounds identified from Cucumis maderaspatanus extracts target AURKA, CDK1, and VEGFR-2 in oral cancer. The most potent compounds, such as Alpha-Curcumene, exhibited strong binding and favorable drug-likeness properties according to Lipinski’s Rule of Five

Similar to CADD-designed peptides, studies on anticancer and anti-inflammatory CADD small molecules in oral research are less prevalent. This finding highlights the research gap and potential value of anticancer and anti-inflammatory small molecules.

#### 3.2.2 Antitumor small molecules

Soykan Agar et al. designed and validated a novel anticancer drug, IHNOCS, through molecular docking and MD simulation for inhibiting head, neck, and oral cancers. IHNOCS targets TGF-β and KRTAP2-3, preventing cancer cell migration while avoiding the uncontrolled proliferation that may result from excessive TGF-β inhibition. The study utilized AutoDock Vina for molecular docking calculations to screen the optimal binding model. Furthermore, Schrödinger’s Desmond was employed to conduct the MD simulation, verifying the stability of the drug‒protein complex. Hydrogen bond mapping analysis further confirmed that IHNOCS remains stable at pH 5.0, the tumor microenvironment and can form stable hydrogen bonds with target proteins. These results indicate that IHNOCS reduces the risk of cancer cell metastasis by partially modulating TGF-β while inhibiting KRTAP2-3. Its targeted action on oral cancer-related proteins suggests its potential therapeutic value for oral cancer treatment (Agar et al., 2024).

#### 3.2.3 Anti-inflammatory small molecules

Pradeep et al. identified key genes and proteins associated with epithelial-mesenchymal transition in Hertwig’s epithelial root sheath, utilizing a systems biology approach, PPI network analysis, and molecular docking techniques. The screening process involves extracting relevant gene and protein lists from the literature, constructing a PPI network via the STRING database, and identifying hub genes within the network (Yadalam et al., 2022). Ultimately, DYRK1A was determined to be a critical target. Modulating DYRK1A expression can attenuate inflammatory responses, providing a foundation for tissue regeneration and repair. Isoetharine was identified as a potential therapeutic candidate through molecular docking and MD simulations and exhibited stable binding with DYRK1A. These findings suggest the potential of Isoetharine as a promising drug candidate for periodontal regeneration (Yadalam et al., 2022).

### 3.3 Plant extracts

Here we synthesize studies where plant extract–based pipelines have already been investigated in oral contexts.

Plant extracts serve as valuable repositories for natural drug development and represent a focal point in preventing and treating oral diseases. By employing CADD techniques such as NP, molecular docking, VS, and MD simulations, the interactions between the active components of plant extracts and their targets can be systematically analyzed. These approaches enable a scientific evaluation of the therapeutic potential of plant extracts in oral applications, including their antibacterial, antitumor, and anti-inflammatory effects (Table 5). Integrating these techniques enhances drug screening efficiency and provides crucial theoretical support for the development of effective and safe plant extract-based oral therapeutic products.

**TABLE 5:** Plant extracts that can be used for oral diseases studied with CADD-associated technology.

Drug name	CADD-associated technology	Main experimental content
Tissue repairing drugs		
Glabridin, baicalin, quercetin (Chen and Song, 2024)	NP was used to screen active compounds and potential targets, constructing a “TCM–Component–Target” network. Cytoscape software was used to analyze the protein‒protein interaction (PPI) network, and GO/KEGG enrichment analysis was performed to verify the mechanism of action	The active ingredients and mechanisms of action of Ganlu Drink in treating recurrent oral ulcers were analyzed. Two hundred seventy-eight potential targets were identified, regulating pathways such as the chemical carcinogenesis receptor activation pathway and the PI3K-AKT signaling pathway
Antibacterial drugs		
Curcumin (Guzmán-Flores et al., 2024)	An integrated NP and molecular docking approach was applied, combining multiple databases to analyze potential targets and metabolic pathways	The molecular mechanisms of curcumin against S.mutans were investigated, revealing its antibacterial effects through regulating inflammation and bacterial metabolic pathways (e.g., fatty acid metabolism and pyrimidine metabolism)
Honeysuckle extract (Liu et al., 2024b)	Secondary metabolites were screened, and AutoDock Vina molecular docking software was used to evaluate the binding energy and mechanism of active compounds	The inhibitory effects of honeysuckle extract on *S.mutans* biofilm were studied, identifying nine compounds that significantly reduced polysaccharide content. Chlorogenic acid inhibits polysaccharide synthesis and sugar metabolism, while oleanolic acid disrupts adhesion and acid tolerance, jointly impairing *S.mutans* biofilm formation
α-Mangostin (Nguyen et al., 2014)	Molecular docking analysis using HEX software was performed to predict the binding sites and mechanisms of α-mangostin with *S.mutans* glucosyltransferases (GtfB and GtfC)	Molecular docking confirmed the inhibitory effect of α-mangostin on *S.mutans* biofilm formation. Experimental validation demonstrated that α-mangostin disrupted biofilm structure, inhibited acid production, and interfered with key enzyme activity
β-Sitosterol (Evangelina et al., 2021)	Molecular docking analysis predicted the antibacterial activity of β-sitosterol as an inhibitor of MurA, MurB, PBP, and SrtA enzymes	Extraction, separation, and spectroscopic techniques were used to identify compounds, followed by *in vitro* antibacterial validation. Molecular docking results indicated that β-sitosterol inhibits peptidoglycan biosynthesis by binding to MurA and SrtA, thereby preventing bacterial cell wall formation
Epigallocatechin Gallate (EGCG) Han et al. (2021) Hairul Islam et al. (2020), Stavroullakis et al. (2021), Xu et al. (2012), Melok et al. (2018)	Molecular docking with AutoDock analyzed EGCG binding to S.mutans glucansucrase, visualized via PyMOL MD simulations assessed its impact on key S.mutans proteins and biofilm formation, while VS using PubChem and RCSB databases identified potential targets	EGCG was found to inhibit *gtf* gene expression and glucosyltransferase activity, reducing biofilm formation. The optimized derivative EGCG-S exhibited enhanced stability and bioavailability, achieving complete inhibition of *S.mutans*
Anti-inflammatory drugs		
Paeoniflorin (Qi et al., 2022)	A combination of NP and molecular docking techniques was employed, utilizing TCMSP and SwissTargetPrediction databases to screen targets. A PPI network was constructed using STRING, followed by GO/KEGG enrichment analysis	The anti-inflammatory effects of paeoniflorin were analyzed, identifying 31 core targets and 33 major signaling pathways (e.g., TNF and IL-17). It was found to inhibit inflammatory cytokines and regulate the MAPK/ERK/p38 pathway
Houttuynia (Wu et al., 2021a)	This study integrates molecular docking and MD simulations to evaluate Houttuynia cordata compounds against key COVID-19 enzymes. Docking analyses using Schrödinger Suite 2020-3 targeted Mpro (PDB ID 6LU7), PLpro (PDB ID 7JRN), and ADRP (PDB ID 6W02)	The anti-inflammatory and antiviral effects of Houttuynia active compounds were investigated. It was found to inhibit viral replication, regulate the NF-κB signaling pathway, and protect lung tissues
Huangbai decoction (Chang et al., 2024)	NP and molecular docking techniques were applied, utilizing the HERB database to screen active components. GeneCards and OMIM databases were used to analyze core targets and pathways	The mechanism of Huangbai Decoction in treating oral lichen planus was studied, and quercetin and other compounds were identified as multitarget regulators of inflammation and immune dysregulation via the PI3K-Akt signaling pathway
*Lithospermum erythrorhizon* (Zhao et al., 2024)	NP and molecular docking methods were used, screening targets through TCMSP and OMIM databases, followed by GO/KEGG functional enrichment and MD simulations	The anti-inflammatory effects of shikonin were studied, and seven key targets and mechanisms of action were revealed such as PI3K-Akt signaling pathway
Verapamil (Wang et al., 2023)	Systems biology, big data mining, and deep learning technologies were employed. A PPI network, gene regulatory network, and DNN-drug target interaction (DNN-DTI) model were used to predict new indications and assess candidate drug sensitivity and toxicity	Drug repurposing for periodontitis treatment was investigated, predicting potential new indications for Verapamil through drug-target interaction analysis, which can reduce of periodontal tissue inflammation
Antitumor drugs		
Apigenin, chrysoeriol, luteolin (G et al., 2024)	Molecular docking was used to screen plant compounds from the PubChem database. AutoDock software was utilized to analyze their binding energy and inhibitory effects on key proteins	Plant compounds were screened to target key proteins in oral squamous cell carcinoma (e.g., Cyclin D1 and PI3K-alpha)
Curcumis maderaspatanus extract (Rao et al., 2024)	Molecular docking and MD simulations were performed to evaluate binding affinity and complex stability, along with pharmacokinetic analysis for drug development potential	Key compounds such as 3-Benzoyl-2,4(1H,3H)-Pyrimidinedione were screened and validated for their binding affinity to oral cancer-related proteins AURKA, CDK1, and EGFR-2, demonstrating potential for drug development
Ginsenoside C and Rg1 (Abouelwafa et al., 2023)	Molecular docking (Schrödinger 12.8) and MD simulation (Demond v.12) were used to evaluate the binding affinity and stability. Protein structures were retrieved from the PDB database, and ligands were selected from DrugBank and preprocessed using LigPrep	Ginsenoside C and Rg1 exhibited high binding scores and stability by interacting with FAP, FN1, and MMP1 gene proteins, suggesting potential as therapeutic agents for oral cancer

Juan et al. explored the molecular mechanisms underlying the therapeutic effects of curcumin on dental caries and its impact on *S. mutans*. By integrating NP and molecular docking techniques, multiple databases (e.g., PubChem, GEO) were utilized to identify potential targets and differentially expressed genes associated with dental caries. Intersection analysis revealed 134 common targets (Guzmán-Flores et al., 2024). Further investigation revealed that curcumin primarily affects seven key proteins, including MAPK1, BCL2, and KRAS, which are closely related to host immunomodulation. Moreover, metabolic network analysis revealed that curcumin influences 11 metabolic pathways, such as fatty acid metabolism, pyrimidine metabolism, and DNA replication in *S. mutans*, thereby inhibiting bacterial growth and survival. These findings suggested that curcumin exerted therapeutic potential in treating dental caries by modulating multiple pathways in both the host and the pathogen (Guzmán-Flores et al., 2024).

Hui et al. employed NP and MD techniques to investigate the anticancer potential of quercetin on oral cancer (Dong et al., 2023). Initially, 190 quercetin-related targets were identified via the TCMSP and SwissTargetPrediction databases, whereas 8971 oral cancer-related targets were obtained from the GeneCards and OMIM databases. Intersection analysis yielded 172 potential targets, which were further screened via a PPI network and Cytoscape software and ultimately identified six core targets: AKT1, PIK3R1, MYC, HIF1A, SRC, and HSP90AA1 (Dong et al., 2023). Molecular docking results demonstrated strong binding affinities between quercetin and these targets, indicating its potential as a multitarget anticancer agent.

Yue et al. investigated the mechanism of baicalein in treating periodontitis through NP, molecular docking, and experimental validation. By integrating data from the TCMSP, SwissTargetPrediction, and GeneCards databases, relevant targets for both baicalein and periodontitis were identified, leading to the selection of 17 core targets, including MMP9, TNF-α, and HIF1A. GO and KEGG analyses revealed that baicalein likely exerted its effects via the MAPK, HIF-1, TNF, and PI3K-Akt signaling pathways. Molecular docking demonstrated favorable binding affinities between baicalein and multiple targets. Experimental validation further confirmed that baicalein significantly reduces the expression of TNF-α and MMP-9 induced by *P. gingivalis*-LPS and inhibits the expression of the M1 macrophage marker iNOS, thereby mitigating inflammatory responses (Liu Y. et al., 2024).

In summary, the application of CADD in oral medicine has made notable progress, encompassing research areas such as oral infections, inflammation, tumors, and tissue regeneration. However, current studies predominantly focus on developing antibacterial agents, whereas research on anti-inflammatory, antitumor, and tissue repair applications remains relatively limited. This disparity suggests that these underexplored areas hold substantial research potential and further developmental value. The uneven distribution of research efforts may be attributed to the varying scope and specificity of medical needs. Compared with oral-specific bacterial infections, such as dental caries and periodontitis, the need for anti-inflammatory, antitumor, and tissue repair interventions extends beyond the oral cavity. They are widely relevant to systemic and other medical subfields. Consequently, research in these domains is often dispersed across broader medical disciplines rather than forming concentrated research hotspots within oral medicine. To systematically explore the potential applications of CADD in oral medicine, we reviewed and synthesized advancements in CADD research related to anti-inflammatory, antitumor, and tissue repair therapies in other medical subfields. By extracting valuable insights from these studies, we aimed to assess their applicability and translational potential in oral medicine.

## 4 Potential CADD-designed drugs for oral diseases

**FIGURE 3:**
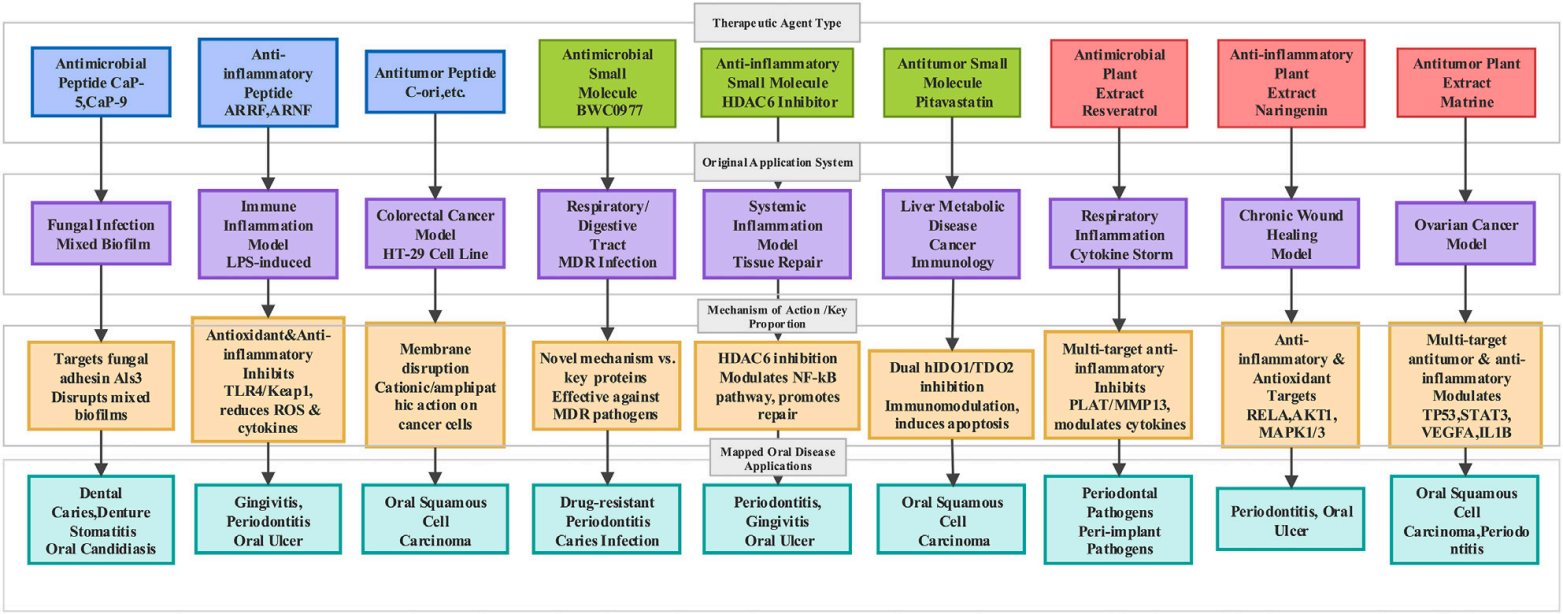
Mapping of therapeutic agents from original applications to oral diseases via mechanistic insights.

As noted earlier, although a number of studies have investigated the use of peptides in oral diseases, important gaps remain. For instance, peptides currently applied to the treatment of oral diseases are largely limited to antimicrobial peptides, with little progress in the development of anti-inflammatory or antitumor peptides. In light of this, we further reviewed studies in which CADD-based drug design has been applied in other areas of medicine, and systematically analyzed potential agents by drawing parallels between these diseases and oral conditions (Figure 3). We hope that the design concepts and scientific methodologies established in other disciplines may provide valuable inspiration for the development of novel therapeutics for oral diseases.

### 4.1 Peptides

CADD-designed peptides have been explored in antimicrobial, anti-inflammatory, and antitumor research fields. Notably, advancements in these interdisciplinary studies have deepened fundamental understanding of biological processes and improved translational value for applications in oral healthcare, including the development of innovative diagnostic tools, targeted therapeutics, and biomaterial-based solutions for treating dental and periodontal diseases (Table 6).

**TABLE 6:** Peptides with potential for oral diseases studied with CADD-associated technology.

Drug name	CADD-associated technology	Main experimental content	Potential as oral medicine
Antibacterial drugs			
DM3 (Le et al., 2015)	ATPs and MD simulations were used to screen and validate effective ATPs	DM3, combined with penicillin, increased the survival rate of infected mice to 100%. It exhibited low toxicity with no significant damage to major organs. Molecular docking analysis revealed strong binding affinity to key proteins of *Streptococcus* pneumoniae (e.g., autolysin)	DM3 targets virulence factors in *Streptococcus pneumoniae* (e.g., autolysin LytA), which are also found in oral bacteria like *S. mutans* and *Actinomyces*, making it potentially effective. It acts on both gram-positive and gram-negative bacteria, fitting the diverse oral microbiome. Its biofilm-disrupting ability helps reduce dental plaque and improve periodontal treatment
MDP1 (Ekhtiari-Sadegh et al., 2024)	Molecular docking and molecular modeling were used to analyze the interaction between MDP1 ATPs and MECs. ChemDraw Suite and I-TASSER optimized the structures, while AutoDock Vina calculated a binding affinity, indicating moderate interaction. Discovery Studio further analyzed interaction types and bond distances	MDP1 hydrogel completely eradicated MRSA and VRSA within 3 h. It exhibited excellent anti-biofilm properties, good biocompatibility with no toxicity to skin cells, and a rapid drug release profile that remained effective across different pH levels	MDP1 hydrogel’s potent anti-biofilm properties and rapid drug release profile make it well-suited for managing oral and postsurgical infection control. Its safety and efficiency support its application in oral medicine
Abaucin (Liu et al., 2023)	Over 7,500 molecules were screened for inhibition of *A. baumannii*, A DMPNN converted molecular structures into vectors, enhanced by RDKit fingerprints for better antibacterial prediction. HTVS of 6,680 compounds from the Drug Repurposing Hub led to the discovery of Abaucin	Deep learning discovered Abaucin, a narrow-spectrum antibiotic targeting *Acinetobacter baumannii*. It inhibits *A. baumannii* by blocking lipoprotein trafficking (LolE pathway) with strong efficacy in both *in vitro* and mouse infection models	Abaucin targets *A. baumannii*, preserving oral microbiota and reducing resistance spread. Its LolE-mediated inhibition may help treat gingivitis and periodontitis while its anti-biofilm potential could prevent plaque and infections. Mouse studies confirm strong antibacterial effects
Halicin (Zhang et al., 2024)	Halicin was discovered using artificial intelligence (AI) for drug screening. AI was used to analyze over 6,000 compounds in the Drug Repurposing Hub, identifying Halicin as a potential broad-spectrum antibacterial agent	*In vitro* tests showed it inhibits *C. perfringens*, and mouse models confirmed its efficacy. Toxicity tests indicated high safety. Pharmacokinetics showed poor absorption, rapid metabolism, and suitability for intestinal infections. It also had minimal gut microbiota impact and recovered faster than ciprofloxacin (CIP)	Halicin’s broad-spectrum antibacterial properties effectively inhibit oral pathogens like *C. perfringens*. It has low toxicity, rapid clearance, and minimal mutagenicity, making it a safe oral candidate. Unlike traditional antibiotics, it resists bacterial resistance and preserves oral microbiota balance
Anti-inflammatory drugs			
ARRF and ARNF (Xin et al., 2024)	Virtual enzymatic digestion technology was used to screen peptide fragments, followed by molecular docking and MD simulations to assess stability. Cellular experiments were conducted to verify functional properties	ARRF and ARNF were identified and found to interact with receptors via hydrogen bonding and hydrophobic interactions. Cellular experiments demonstrated that these peptides inhibited inflammatory cytokine secretion, reduced oxidative stress levels, and enhanced antioxidant enzyme activity, exhibiting significant anti-inflammatory and antioxidant effects	ARRF and ARNF’s anti-inflammatory and antioxidant properties make them suitable for preventing oral inflammatory diseases such as gingivitis, periodontitis, and oral ulcers. Their low toxicity and high safety profile make them ideal candidates for oral health products such as mouthwash and toothpaste
Anti-tumor drugs			
Cordyceps militaris (Chantawannakul et al., 2021)	The study utilized machine learning models (AntiCP, iACP, and MLACP) to screen for anticancer peptides (ACPs) and assessed their toxicity and cell penetration capability using ToxinPred and MLCPP. Furthermore, AntiCP was used to optimize anticancer properties, while PEP-FOLD3.5 molecular modeling was applied to refine physicochemical properties	In HT-29 colorectal cancer cell line experiments, C-ori selectively bound to and disrupted cancer cell membranes due to their cationic nature and amphipathic structure, leading to apoptosis and significantly inhibiting tumor proliferation	Given the similarity in membrane charge and biochemical properties between oral and colorectal cancer cells, C-ori may exhibit similar anticancer effects in oral cancer treatment. Additionally, localized drug delivery systems (such as oral patches or gels) could enhance therapeutic efficacy and enable precise treatment

#### 4.1.1 Antibacterial peptides

##### 4.1.1.1 CaP-5, CaP-9

Zhang et al. initially employed CADD technology to screen a series of short cationic peptides targeting *C. albicans* infection, and evaluated their stability and safety using AntiBP2 and ToxinPred. Molecular docking and dynamics simulations showed strong binding of these peptides to the fungal cell wall adhesin Als3, while experimental validation confirmed that CaP-5 and CaP-9 significantly inhibited *C. albicans* adhesion and biofilm formation, and were also effective against mixed biofilms of *C. albicans* and *S. mutans*. Since common oral infections such as caries, denture stomatitis, and oral candidiasis are closely associated with mixed biofilms, these peptides demonstrate the ability to act on both fungi and bacteria, disrupt complex microbial communities, and thus hold strong potential for application in oral antimicrobial therapy (Awdhesh Kumar Mishra and Kodiveri, 2024).

#### 4.1.2 Anti-inflammatory peptides

##### 4.1.2.1 ARRF and ARNF

ARRF and ARNF were selected via ExPASy Peptide Cutter software, which was employed to simulate the enzymatic actions of trypsin, pepsin, and papain, generating 529 peptide fragments. The biological properties of these peptides were further validated via molecular docking and MD simulation techniques, revealing that hydrogen bonding and hydrophobic interactions play crucial roles in their binding to the Keap1 and TLR4 receptors, with high stability in receptor binding. Experimental validation demonstrated that ARRF and ARNF exhibited significant antioxidant and anti-inflammatory activities in LPS-induced RAW264.7 macrophage models. Specifically, these peptides suppressed the TLR4 signaling pathway, reducing the secretion of inflammatory cytokines such as TNF-α and IL-6. Moreover, they alleviated oxidative stress by decreasing reactive oxygen species (ROS) levels and increasing the activities of antioxidant enzymes, including SOD and GSH-Px. Compared with conventional peptide screening methods, this study significantly improved screening efficiency by integrating virtual enzymatic hydrolysis with molecular simulation techniques. Their anti-inflammatory properties may help mitigate gingivitis and other oral inflammatory conditions. Moreover, their antioxidant effects may offer protective benefits in treating oxidative stress-related oral diseases such as oral ulcers and periodontitis. Additionally, naturally derived functional peptides from food sources exhibit low toxicity and high safety, making them suitable for development into oral health products such as mouth rinses or toothpaste (Xin et al., 2024).

#### 4.1.3 Anti-tumor peptides

##### 4.1.3.1 Cordyceps militaris

AMPs with anticancer potential derived from Cordyceps militaris were screened via pepsin digestion simulations to generate 21,148 peptide sequences, which were then screened for anticancer activity via 3 ML models—AntiCP, iACP, and MLACP. Toxicity and cell penetration capabilities were assessed via ToxinPred and MLCPP to ensure high efficiency and safety. The screened AMPs were further optimized through AntiCP, and their physicochemical properties were refined via PEP-FOLD3.5 molecular modeling. In HT-29 colorectal cancer cell line experiments, the AMPs selectively bound to and disrupted cancer cell membranes due to their cationicity and amphipathic structure, thereby inducing apoptosis and inhibiting tumor proliferation. Notably, given the similarity in membrane charge and biochemical properties between oral and colorectal cancer cells, C-ori may exhibit similar therapeutic effects in oral cancer treatment. Moreover, localized drug delivery systems, such as oral patches or gels, could be utilized for effective therapeutic administration (Zhuang and Ibrahim, 2021).

### 4.2 Small molecules

CADD-designed small molecules have been explored for their antibacterial, anti-inflammatory, and anticancer effects. Moreover, advancements in these disciplines have translational potential for applications in oral health (Table 7).

**TABLE 7:** Small molecules with potential for oral diseases studied with CADD-associated technology.

Drug name	CADD-associated technology	Main experimental content	Potential as oral medicine
Antibacterial drugs			
DNA Gyrase B (GyrB) Inhibitors (Lian et al., 2021)	Chemical synthesis combined with ligand-based virtual screening (LBVS) were used to evaluate the antibacterial activity of novel GyrB inhibitors	A total of 18 2,3-diaminoquinoxaline derivatives were chemically synthesized, and LBVS identified potent GyrB inhibitors (e.g., AG-205 and 6c). Experimental results demonstrated significant antibacterial activity against MRSA (MIC values of 4–8 μg/mL and 0.5 μg/mL, respectively) by inhibiting DNA supercoiling	The high antibacterial efficacy of GyrB inhibitors against MRSA strains suggests a new therapeutic approach for soft tissue infections in the oral cavity. Potential applications include the development of oral gels targeting drug-resistant infections, with a focus on their effectiveness in soft tissue infections
Novel DNA Gyrase Inhibitors (Sterle et al., 2023)	Schrödinger Glide XP were employed for molecular docking to investigate the binding interactions of DNA gyrase (GyrB) inhibitors and their siderophore mimic conjugates. The docking simulations were conducted based on the crystal structure of *E. coli* GyrB (PDB: 4DUH) to optimize inhibitor design and enhance antibacterial efficacy​	A total of 23 novel DNA gyrase inhibitors were designed and synthesized based on molecular docking and structural optimization. Compounds 34 and 35 exhibited potent inhibition of *E. coli* DNA gyrase (IC50 = 0.058 µM and 0.11 µM, respectively). By leveraging SBDD and targeting DNA supercoiling, these inhibitors demonstrated broad-spectrum antibacterial activity against oral pathogens	DNA gyrase inhibitors, by blocking DNA supercoiling, exhibit broad-spectrum antibacterial potential in treating antibiotic-resistant oral pathogens. Further optimization of permeability and stability is needed, along with investigations into their application in treating multidrug-resistant oral infections. The incorporation of iron carrier strategies could enhance specificity and efficacy, facilitating the development of novel antibacterial agents
Lpl-004, Lpl-008, Lpl-023 (Scattolini et al., 2024)	SBVS, molecular docking, and gene editing were used to construct bacterial knockout models for mechanism and antibacterial activity assessment	Lpl-004 interferes with the E2 subunit of the fatty acid-dependent enzyme complex, inhibiting metabolic functions and virulence expression in *S. aureus*, making it suitable for treating resistant bacterial infections	Lpl-004 and its analogs could be developed for the treatment of *S.aureus*-related oral infections
Pleuromutilin derivatives (Wu et al., 2022)	Molecular docking (AutoDock Vina) analyzed urea derivatives binding to the 50S ribosome (*D. radiodurans*, PDB: 1XBP). ADMET prediction (admetSAR, ADMETlab) assessed pharmacokinetics and toxicity	Pleuromutilin derivatives, such as 6 m and 6n, exhibited highly potent antibacterial activity against gram-positive bacteria, with MIC values as low as 0.0625 μg/mL and low toxicity	Pleuromutilin derivatives could be formulated to treat gram-positive bacterial infections in the oral cavity
Terpenoid compounds (Daisy et al., 2008) (Extracted from *Elephantopus scaber*)	PASS prediction and molecular docking technology targeting autolysins were used to predict terpenoid activity	Terpenoid compounds extracted from Elephantopus scaber inhibited cell wall degradation by binding to autolysins and were effective against drug-resistant *S. aureus*	Terpenoid compounds are suitable for oral antibacterial formulations, potentially preventing S.aureus-related oral infections
Heme oxygenase inhibitors (Furci et al., 2007)	CADD virtual screening, molecular docking, and two-round selection were used to identify heme oxygenase inhibitors, followed by *in vitro* experimental validation	Identified heme oxygenase inhibitors effectively blocked bacterial iron acquisition, inhibiting the growth and biofilm formation of *Pseudomonas aeruginosa*	Heme oxygenase inhibitors reduce oral pathogen survival by limiting iron availability, making them promising candidates for preventing and treating biofilm-related oral infections
Luteolin, kaempferol, and curcumin (Nandhini et al., 2023)	Molecular docking and MD simulation combined with StarDrop and Desmond software were used to evaluate binding energy and pharmacokinetic properties	Natural compounds such as luteolin exhibit significant antibacterial activity against MRSA, disrupting cell walls and inhibiting key enzyme activities	Due to their anti-inflammatory properties, these natural compounds are suitable for oral infection and inflammation
Trimethrexate (#66) (Zhang et al., 2015)	This study optimized DHFR inhibitors using homology modeling, molecular docking, and binding energy analysis. A S.mutans DHFR model was validated based on *S. pneumoniae* DHFR. Docking calculations assessed inhibitor binding affinity to S.mutans and human DHFR.	Based on the FDA-approved drug trimetrexate (TMQ), computational drug design techniques identified a potent inhibitor of S.mutans DHFR, compound #66 (IC50 = 8.7 nM), exhibiting a selectivity index of 117.8 for human DHFR.	They effectively inhibit *S. mutans* growth and biofilm formation while having minimal impact on human DHFR (SI = 117.8) and oral commensal bacteria. With a precise mechanism targeting folate metabolism, these compounds can be further optimized, making them promising candidates for dental caries prevention and treatment
Benzoylaminobenzoic Acid Derivatives (Drug Repurposing) (Singh et al., 2008)	QSAR Analysis and Hansch and Free-Wilson Methods were used to evaluate the inhibitory activity	A total of 46 compounds were screened, and their inhibitory activity was enhanced by increasing hydrophobicity, aromaticity, and hydroxyl groups; the inhibitory effect on FabH was validated, demonstrating significant antibacterial activity against pathogens such as S.aureus and E.coli	The broad-spectrum antibacterial effects suggest the potential application in inhibiting oral pathogens
Semi-Synthetic Amoxicillin-Derived Azomethine Derivatives (Drug Repurposing) (Mullasseril, 2014)	Through molecular modeling and Lipinski’s rule screening, pharmacokinetic properties were ensured. Then, molecular docking was performed using ArgusLab4.0.1 to analyze the binding affinity with β-lactamase and evaluate antibacterial activity	The D1S1 and D1S3 derivatives exhibited superior antibacterial activity compared to the parent drug, amoxicillin; QSAR endpoint predictions indicated low toxicity, compliance with Lipinski’s Rule, and potential drug activity	The derivatives demonstrated significant antibacterial activity against drug-resistant oral bacteria, suggesting potential as a novel antimicrobial agent for oral disease prevention and treatment
Anti-inflammatory drugs			
HDAC6 Inhibitors (A1 and B1) (Dawood et al., 2020)	The study combined CADD virtual screening and molecular docking and used PyRx and AutoDock to optimize screening parameters, validating HDAC6 inhibitors	A1 and B1, as HDAC6 inhibitors, not only exhibited significant anti-tumor activity in leukemia cells but reduced the release of inflammatory cytokines such as IL-6, TNF-α, and IL-1β, thereby decreasing inflammatory responses	By reducing inflammatory cytokine levels, A1 and B1 hold the potential to alleviate periodontal tissue inflammation and promote oral tissue regeneration
M20 (Song et al., 2021)	The study employed SBVS combined with Glide SP/XP molecular docking for compound screening	M20 effectively inhibited MyD88 homodimerization, significantly reducing LPS-induced TNF-α and IL-6 release, and demonstrated strong anti-inflammatory effects in a murine inflammatory lung injury model	The anti-inflammatory properties of M20 can be applied to treat oral inflammation-related diseases such as periodontitis by suppressing inflammatory cytokine release and the inflammatory cascade reaction
Indomethacin (Ali et al., 2023)	The study combined experimental and computational methods, including UV absorption spectroscopy, fluorescence spectroscopy, molecular docking, and MD simulation	Indomethacin exhibited strong binding stability with HSA, effectively inhibiting COX enzyme activity, reducing prostaglandin production, and alleviating inflammation, fever, and pain	Indomethacin can be further developed into an oral anti-inflammatory analgesic for relieving symptoms of periodontitis, gingivitis, and oral ulcers, improving patients’ quality of life
Tanshinone IIA (TAS) (Liu et al., 2024a)	TAS was identified through VS from the TCMSP database, and its binding to ESRRG was analyzed using MD simulation	TAS reduced oxidative stress levels by modulating the ESRRG pathway, significantly decreasing apoptosis and mitochondrial damage, improving inflammatory markers, and enhancing tissue health	Through its antioxidant and anti-inflammatory properties, TAS can be developed into herbal formulations suitable for the oral environment, protecting gingival health and promoting tissue repair
C29 and Vanillin (Mistry et al., 2015)	The study utilized CADD to analyze and screen the BB loop pocket structure in TLR2 protein	C29 and vanillin inhibited TLR2 signaling pathways both *in vitro* and *in vivo*, reducing the release of inflammation-related factors and significantly alleviating TLR2-mediated inflammatory responses	C29 and vanillin can be used to control oral inflammatory diseases, mitigate tissue destruction caused by TLR2 signal activation, and explore their potential applications in oral cancer
Chlorhexidine O-GlcNAcase Inflammatory Regulation (Drug Repurposing (Dong et al., 2019)	VS from DrugBank, molecular docking, and MD simulation were used for drug design and activity prediction	Chlorhexidine effectively inhibits O-GlcNAcase, significantly reducing inflammatory factor production, playing a crucial role in cellular repair and metabolism	Exploiting chlorhexidine’s anti-inflammatory effects to develop oral anti-inflammatory formulations, and investigating its combined effects on tissue repair
GF-17 (Aghazadeh et al., 2020)	MD, the potential of mean force (PMF) calculations, and the CHARMM36 force field were employed to study the interactions between the ABPs and bacterial membranes	MD simulations examined GF-17’s interaction with bacterial membranes, comparing DPPE/DPPG (gram-negative) and DPPG (gram-positive) bilayers. GF-17 penetrates DPPG more efficiently, increasing lipid area and diffusion. It disrupts DPPG via toroidal pores and DPPE/DPPG via a carpet model, highlighting its antibacterial potential	GF-17, derived from LL-37, shows vigorous antimicrobial activity, particularly against gram-positive bacteria involved in oral diseases like periodontitis. LL-37 has known anti-inflammatory and wound-healing properties, suggesting GF-17 might also help reduce inflammation and promote healing in oral conditions such as gingivitis, periodontitis, and oral ulcer
Anti-tumor drugs			
Bexarotene and Oxymorphone (Shahab et al., 2023)	SBVS, molecular docking, MD simulation, and MMGBSA free energy calculation were used for drug design, optimization, and activity prediction	Using the DrugRep platform and molecular docking and dynamics simulation, VS identified Bexarotene and Oxymorphone as HDAC6 and VISTA inhibitors, respectively. Bexarotene exhibited a low binding free energy (−51.97 kcal/mol), while Oxymorphone had a binding free energy of −36.83 kcal/mol for VISTA. Both showed high binding stability and potential in immune microenvironment modulation	The combined application of Bexarotene and Oxymorphone in oral squamous cell carcinoma therapy may optimize dosage and administration, alleviate immune suppression, enhance T-cell cytotoxicity, and improve survival rates in advanced oral cancer patients.
ZINC12143050, ZINC08301324, ZINC16743012, and ZINC64165826 (Molla et al., 2023)	Pharmacophore modeling, molecular docking, and MD simulation were used for MAOB inhibitors	Pharmacophore modeling generated an MAOB active site model for VS of a compound library. Four candidate compounds (ZINC12143050, ZINC08301324, etc.) were identified and validated for high binding affinity and stability (binding scores ranging from −11.7 to −11.1 kcal/mol) through docking and MD simulation. These compounds modulate reactive oxygen species levels and induce apoptosis, potentially improving the oral cancer tumor microenvironment	These molecules reduce oxidative stress in the tumor microenvironment of oral cancer by targeting the MAOB protein. Further drug modifications aim to minimize toxicity and develop a low-toxicity therapeutic strategy for oral cancer treatment
HS-1, HS-2, HS-3, and HS-4 (Yang et al., 2023b)	Dual-target inhibitors of HDAC1 and SPOP were identified through SBVS and molecular docking, followed by MD simulations for validation	From a library of 43,000 compounds, HS-1 to HS-4 were identified as dual-target inhibitors of HDAC1/SPOP. Among them, HS-2 exhibited the most potent inhibition with IC50 values of 7.6 nM for HDAC1 and 9.1 µM for SPOP. MD simulations and *in vitro*/*in vivo* studies confirmed strong antitumor efficacy	HS-2 shows strong potential as an oral anticancer agent by dual inhibiting HDAC1 and SPOP. It effectively suppresses cancer cell proliferation, outperforming reference drugs with IC50 values of 7.6 nM (HDAC1) and 9.1 μM (SPOP). MD confirms stable binding, and *in vivo* studies demonstrate significant tumor suppression with minimal toxicity
66 Pyrazolo[3,4-d]pyridazinone (Wu et al., 2021b)	A combination of molecular docking, pharmacophore modeling, MD simulations, and free energy calculations was utilized. Multistep chemical synthesis introduced various substituents to optimize activity, with structural characterization via NMR and HRMS.	66 pyrazolo [3,4-d]pyridazinone derivatives were synthesized and structurally characterized. The lead compound, 10 h, demonstrated superior FGFR inhibitory activity, effectively suppressing tumor angiogenesis and cell proliferation, highlighting its potential for oral cancer therapy	Combining FGFR inhibitors with existing chemotherapy or immunotherapy can target angiogenic tumors in oral cancer
Pitavastatin (Aboomar et al., 2024)	A VS approach integrating molecular docking and MD simulations was employed to develop a ligand-based pharmacophore model for dual enzyme inhibition	Through VS and MD simulations, Pitavastatin was identified as a dual inhibitor of hIDO1 and hTDO2. *In vitro* validation revealed its ability to induce apoptosis in oral cancer cells by downregulating IL-6, STAT3, and AhR signaling, leading to caspase-3 activation. It also exhibited potential for modulating the tumor immune microenvironment with favorable safety profiles	By inhibiting hIDO1 and hTDO2 enzymes, Pitavastatin prevents the formation of immunosuppressive metabolites, thereby weakening the immunosuppressive tumor microenvironment. This enhances T-cell-mediated cancer cell elimination via caspase-3 activation, making it a promising candidate for oral squamous cell carcinoma treatment
Temozolomide (TMZ) Derivatives S1, S3, S8, and S10 (Drug Repurposing) (Qian and Zhu, 2018)	A 3D structure-activity relationship model was constructed using CADD, molecular docking, and ADME/toxicity predictions to optimize the derivatives	Derivatives S1, S3, S8, and S10 significantly inhibited the growth of U87MG and U251 glioblastoma cells by targeting AGPS. These derivatives improved TMZ’s absorption, distribution, metabolism, and excretion (ADME) properties, reduced toxicity, and enhanced antitumor efficacy	The potential of their application in oral diseases may improve by optimizing the drug delivery systems for S1, S3, S8, and S10 to enhance their suitability for oral cancer treatment and exploring their anti-inflammatory effects on periodontitis or other oral inflammation
Tucatinib-Based HER2 PROTAC Degrader CH7C4 (Drug Repurposing) (Hu et al., 2022)	CADD, molecular docking, and PROTAC design were used for drug design, optimization, and activity prediction	Optimization of PROTAC E3 ligase ligand, linker length, and tucatinib derivative site resulted in forming an effective target protein-PROTAC-E3 complex, potentially valuable for HER2-positive cancer therapy	CH7C4 is the first highly effective and selective HER2 degrader. Compared to traditional HER2-targeted drugs like Tucatinib, it completely degrades HER2, preventing drug resistance and inhibiting key oncogenic pathways (PI3K/AKT, RAS/RAF/ERK). Since some oral squamous cell carcinomas (OSCC) overexpress HER2, CH7C4 may effectively treat these tumors

#### 4.2.1 Antibacterial small molecules

The Bugworks Research Team screened a chemical library comprising approximately 3,000 compounds from commercial databases such as Molecule and Enamine. Initial phenotypic screening was performed using an *E. coli* model, followed by structural optimization of the identified lead compounds. MD simulations and SAR analyses were employed to refine the compound’s binding mode to the target proteins during the drug optimization process. BWC0977 demonstrated potent antibacterial activity, with a minimum inhibitory concentration ranging from 0.03 to 2 μg/mL against a broad spectrum of MDR pathogens. Additionally, its ability to achieve high drug concentrations in infected tissues, such as pulmonary epithelial lining fluid, suggests that it may offer similar advantages in treating localized tissue infections, including oral infections. This makes it particularly suitable for combating MDR strains in oral infections (Hameed et al., 2024).

#### 4.2.2 Anti-inflammatory small molecules

By using CADD techniques in conjunction with VS and molecular docking, two potential HDAC6 inhibitors with anti-inflammatory properties were identified and validated, A1, 5-(4-bromonaphthalene-1-sulfonamido)-2-hydroxybenzoic acid, and B1, N-(9-oxo-9H-fluoren-3-yl)-benzamide. The study screened over 175,000 compounds from the ZINC15 and OTAVA chemical databases via PyRx software to identify candidates with high binding affinity. This step was followed by further molecular docking optimization to confirm the reliability of their interactions with HDAC6. A1 and B1 effectively downregulate inflammatory cytokines such as IL-6, TNF-α, and IL-1β while suppressing key inflammatory pathways, including the NF-κB pathway. These compounds may demonstrate significant anti-inflammatory effects in treating periodontitis and gingivitis by mitigating inflammatory responses, inhibiting alveolar bone resorption, and promoting periodontal tissue repair. Additionally, HDAC6 inhibitors have been shown to play crucial roles in cell migration and tissue regeneration, suggesting their potential to enhance the healing of oral ulcers (Dawood et al., 2020).

#### 4.2.3 Anti-tumor small molecules

Notably, both A1 and B1 can also induce apoptosis in leukemia cells, including multidrug-resistant CEM/ADR5000 cell lines, indicating their potential anticancer activity and offering a promising avenue for oral cancer treatment (Dawood et al., 2020).

A study utilized VS and MD simulations to confirm the potent dual inhibitory effect of Pitavastatin on hIDO1 and hTDO2. Researchers performed molecular docking to screen potential inhibitors from the DrugBank database and found that Pitavastatin exhibited high binding affinity for both targets. Subsequently, MD simulations were conducted via GROMACS to assess its stability. The simulations employed the GROMOS 54A7 force field. The TIP3P water model was used for solvation, and the PME method was applied for electrostatic calculations. Pitavastatin effectively reduced immune suppression, enhancing immune-mediated clearance of tumor cells. Additionally, Pitavastatin induced G1/S cell cycle arrest, activated the Caspase-3 pathway to promote apoptosis in cancer cells, and downregulated key oncogenic and inflammatory signaling molecules, including STAT3, AhR, and IL-6, thereby suppressing tumor invasion. Given its immunomodulatory, anti-inflammatory, and antitumor effects, Pitavastatin is a promising candidate for oral cancer therapy, paving the way for novel treatment strategies (Aboomar et al., 2024).

### 4.3 Plant extracts

Here we emphasize extra-oral pipelines and mechanisms that could translate to oral indications. CADD technologies have been widely applied to study the anti-inflammatory and antitumor properties of plant extracts. Furthermore, advancements in these interdisciplinary research areas hold significant translational potential for applications in oral health (Table 8).

**TABLE 8:** Plant extracts with potential for oral diseases studied with CADD-associated technology.

Drug name	CADD-associated technology	Main experimental content	Potential as oral medicine
Anti-inflammatory drugs			
Apigenin (Tang et al., 2024)	The study employed HTVS to identify the flavonoid apigenin from over 19,000 compounds. MD simulation, fluorescence detection, and other techniques were used for further validation	Apigenin exhibited significant antibacterial properties, enhancing colistin’s efficacy against gram-negative bacteria. It interfered with the thermal stability of the mcr-1 protein, suppressing its gene overexpression and disrupting bacterial membrane permeability. Additionally, it inhibits ATP generation and induces nitric oxide (NO) and reactive oxygen species (ROS) production, further strengthening antibacterial activity. Moreover, it significantly reduces the production of inflammatory factors TNF-α and IL-1β, demonstrating pronounced anti-inflammatory properties and favorable pharmacokinetic characteristics	Apigenin’s combined antibacterial and anti-inflammatory properties provide a novel therapeutic approach for infected inflammatory diseases. It holds great potential in controlling antibiotic-resistant strains, reducing antibiotic usage when combined with therapy, lowering resistance risks, and enhancing clinical treatment efficacy
Paeoniflorin (Qi et al., 2022)	The study employed NP methods combined with molecular docking to validate paeoniflorin’s binding affinity to key targets (e.g., MMP 9), constructing a target–compound network to analyze its anti-inflammatory mechanisms	NP analysis identified 863 inflammation-related targets, with molecular docking confirming inhibitory effects on MMP 9. Paeoniflorin exhibited multitarget and multipathway anti-inflammatory effects, particularly in TNF and IL-17 signaling pathways. Molecular docking confirmed its strong binding affinity with core targets, demonstrating the potential for inflammation suppression and providing a theoretical foundation for drug development	Paeoniflorin’s multitarget regulatory properties make it a promising candidate for treating oral inflammatory diseases such as periodontitis. Modulating inflammatory cytokines alleviates oral inflammation and may serve as a potential ingredient for oral healthcare product development
Ailanthone (Ma et al., 2025)	The study integrated NP and cellular experiments to identify active components in Ailanthone extract. Protein‒protein interaction (PPI) network analysis determined key targets such as STAT3 and MAPK1, which were further validated by molecular docking	NP and molecular docking studies revealed that Ailanthone extract exhibited potent inhibitory effects on colorectal cancer cells, particularly by promoting apoptosis. It effectively suppressed cancer cell viability and migration, regulated key pathways such as STAT3 and PI3K-Akt, and induced apoptosis, showcasing its potential in cancer therapy	With its multitarget anti-inflammatory properties, Ailanthone holds promise for treating oral inflammatory diseases, potentially exerting its effects by inhibiting key inflammatory pathways such as the PI3K/Akt and STAT3 signaling pathways, modulating oxidative stress responses, and promoting apoptosis in inflammatory cells
Danshen and Dalbergia Odorifera (Zhao et al., 2022)	The study applied NP and molecular docking to identify active compounds in Danshen and Dalbergia Odorifera and study their therapeutic effects on myocardial infarction (MI)	Active compounds such as carnosic acid and naringenin demonstrated therapeutic effects on MI, inflammation, angiogenesis, and oxidative stress. Carnosic acid downregulated PTGS2 to suppress inflammation, while naringenin upregulated KDR to promote angiogenesis	Danshen and Dalbergia Odorifera possess anti-inflammatory and angiogenesis-promoting effects, making them potential candidates for managing oral diseases like periodontitis. Their local application or oral formulations may offer novel therapeutic solutions for oral disease treatment and tissue repair
Resveratrol (Xiao et al., 2021)	The study utilized NP, PPI network construction, and molecular docking combined with GO and KEGG pathway analysis	The study identified 235 resveratrol-related targets and 510 COVID-19 differential genes. Resveratrol regulates inflammation via IL-17, NF-κB, and TNF signaling pathways, mitigating cytokine storms and acute respiratory distress syndrome	Resveratrol’s anti-inflammatory and antioxidant properties can alleviate periodontal disease-related inflammation, facilitate tissue repair, and reduce infection risks, making it applicable for oral inflammation and tissue regeneration
Andrographolide (Jain and Sudandiradoss, 2022)	The study Applied virtual screening, molecular docking, MD simulations, and ADMET pharmacokinetic analysis to optimize derivatives	The study screened 237 derivatives, identifying Ana2 as a lead compound. Ana2 binds to NF-κB p50 subunit (Cys62), effectively reducing inflammation. MD simulations confirmed stable binding, and ADMET analysis validated its low toxicity and favorable absorption properties	Ana2, as a plant-derived low-toxicity anti-inflammatory agent, can alleviate chronic oral inflammation such as periodontitis
Naringin (Sun et al., 2023)	NP screening identified relevant targets, with molecular docking validating compound–target interactions	The study identified 163 naringin-associated targets. At low concentrations, naringin reduced ROS production and significantly inhibited inflammation	Naringin’s antioxidant and anti-inflammatory properties make it suitable for treating chronic oral inflammations such as periodontitis. Its collagen remodeling ability suggests potential applications in oral soft tissue repair
Forsythoside A (Fu et al., 2023)	The study applied NP analysis, PPI and compound–target networks, and molecular docking	NP analysis constructed a PPI network revealing that Forsythoside A significantly reduced inflammatory cytokines (TNF-α, IL-1β, IL-6) by blocking the TLR4/MYD88/NF-κB signaling pathway. It also decreased collagen deposition and fibrosis severity in bile duct-ligated mice	Forsythoside A’s anti-inflammatory and anti-fibrotic properties make it an effective treatment for periodontitis by modulating cytokine release, reducing inflammatory cell infiltration, and promoting postsurgical periodontal tissue recovery
Ursolic Acid (Yang et al., 2022)	NP analysis combined with PPI network analysis and Cytoscape software was used to screen key targets, followed by molecular docking to evaluate binding capacity	Ursolic acid was found to target the TNF signaling pathway and PI3K/Akt pathway, reducing the expression of inflammatory cytokines and promoting angiogenesis. Molecular docking studies revealed a strong binding affinity	Ursolic acid reduces the expression of oral inflammatory factors (e.g., IL-6 and TNF-α), promotes soft and hard tissue repair, and controls pathogenic infections, demonstrating multiple therapeutic effects in periodontal diseases and mucositis
Isolongifolene Derivatives (Jiang et al., 2024)	ADME modeling was used to predict drug properties, molecular docking, and MD for target binding affinity analysis	The derivative P129 was validated through molecular docking to exhibit high binding affinity with CDK-2. MD simulations confirmed the stability of the complex. Cellular experiments demonstrated that P129 inhibited glioblastoma cell proliferation and migration while inducing apoptosis via the mitochondrial-dependent pathway and downregulating Bcl-2 expression	P129s anti-proliferative and pro-apoptotic properties suggest potential applications in oral cancer treatment. Its molecular stability and low toxicity make it a promising candidate for the development of oral medications targeting rapidly proliferating lesions
Dracocephalum Moldavica L. (Zheng et al., 2024)	Active compound was screened using TCMSP, SBDS, and LBDS, followed by component-target network construction PPI network and signaling pathway analysis	The total flavonoids of Dracocephalum Moldavica L. (TFDM) inhibit NOX-4, reducing ROS generation and activating the AMPK/SIRT1/PGC-1α pathway, which significantly decreases oxidative stress and apoptosis. TFDM effectively protects cardiomyocytes from damage in OGD/R models	TFDM alleviates periodontal inflammation by reducing oxidative stress and apoptosis while protecting periodontal tissues from further damage. Its cytoprotective effects suggest potential applications in postsurgical tissue repair and regeneration
Triptolide Zhang et al. (2022)	NP, PPI network analysis, animal experiments molecular docking and dynamic simulation were conducted to investigate target mechanisms	The derivative P129 was validated through molecular docking to exhibit high binding affinity with CDK-2. MD simulations confirmed the stability of the complex. Cellular experiments demonstrated that P129 inhibited glioblastoma cell proliferation and migration while inducing apoptosis via the mitochondrial-dependent pathway and downregulating Bcl-2 expression	P129s anti-proliferative and pro-apoptotic properties suggest potential applications in oral cancer treatment. Its molecular stability and low toxicity make it a promising candidate for developing oral medications targeting rapidly proliferating lesions
Anti-tumor drugs			
Matrine Chen and Song (2024)	NP and Mendelian randomization (MR) analysis combined with molecular docking was used to identify key targets and validate anticancer mechanisms	Six core targets (TP53, CCND1, IL-1β, etc.) were identified. Molecular docking and *in vitro* experiments confirmed that matrine upregulates TP53 while downregulating CCND1 and IL1B, effectively inhibiting cancer cell proliferation and migration while mitigating inflammatory responses	By modulating IL1B and TP53 signaling, the machine can be an adjunct therapy for oral cancer while alleviating oral inflammation
Brucein A Nandana et al. (2023)	NP and PPI network analysis was used to screen cancer-related key genes, followed by molecular docking to simulate target protein interactions	NP identified 105 key targets. Bruce A was validated to modulate TP53 and STAT3, inducing autophagy and apoptosis. Molecular docking confirmed high binding energy with TP53, and experimental results supported its antitumor activity	Brucein A has potential applications in oral cancer treatment by inducing cancer cell apoptosis and reducing inflammation, thereby alleviating oral tissue damage
Paclitaxel Derivatives Rathaur et al. (2021)	NP was used to construct a compound-disease-target (C-D-T) network, combined with molecular docking, MD simulation, and ADME analysis	The study identified natural compounds (e.g., bergenin and silymarin) that synergize with paclitaxel by inhibiting VEGFR2, significantly enhancing anticancer effects. Experiments confirmed reduced tumor angiogenesis and validated *in vivo* and *in vitro* efficacy against metastatic breast cancer	Stabilizing microtubules and blocking VEGFR2 signaling effectively limit tumor growth and metastasis. Their potential for localized delivery in the oral cavity enhances efficacy while minimizing systemic side effects
Tissue repair			
Icariin Long et al. (2020)	PharmMapper-based target prediction, PPI network analysis, and GO/KEGG functional pathway enrichment analysis were used to predict the function	Icariin was found to regulate the Wnt/β-catenin and RANKL/RANK/OPG signaling pathways, promoting osteogenesis, increasing bone mineral density and serum estrogen levels in ovariectomized rats, while significantly reducing bone resorption and osteoporosis risk	Icariin may enhance alveolar bone regeneration and osteoblast differentiation, making it suitable for peri-implantitis and bone defect repair, as well as orthodontic adjunct therapy and periodontal treatment

#### 4.3.1 Antimicrobial plant extracts

A study explored the potential of resveratrol in alleviating COVID-19-related inflammation through NP methods. Using the TargetNet and Comparative Toxicogenomics Database, 235 resveratrol-associated targets were identified, and 510 differentially expressed genes related to COVID-19 were screened via the GEO dataset. Through PPI network and molecular docking analyses, resveratrol was verified to have strong binding affinity with key targets such as PLAT and MMP13. GO and KEGG pathway analysis revealed that resveratrol regulated inflammation via the IL-17, NF-κB, and TNF signaling pathways, potentially mitigating cytokine storms and acute respiratory distress syndrome. These findings highlight the anti-inflammatory and antimicrobial properties of resveratrol, suggesting its potential application in oral health. Its antioxidant and antifibrotic properties may contribute to oral tissue repair (Xiao et al., 2021).

#### 4.3.2 Anti-inflammatory plant extracts

An integrated approach, including NP, molecular docking, and *in vitro*/*in vivo* experiments, was used to investigate the mechanism of naringenin in chronic wound healing. Molecular docking analysis was conducted via CB-Dock2 to identify binding pocket sites, calculate docking scores, and predict binding modes. 163 related targets were identified, which are associated primarily with oxidative stress, inflammation regulation, and metabolic processes and have significant anti-inflammatory and antioxidant effects. The key targets included RELA, AKT1, MAPK1, and MAPK3. These targets are considered crucial for the therapeutic effects of naringenin in chronic wound healing. Molecular docking results indicated that naringenin could bind stably to these targets. In cellular experiments, low concentrations of naringin reduced ROS production and inhibited the expression of inflammatory cytokines such as TNF-α and IL-6 (Sun et al., 2023).

#### 4.3.3 Antitumor plant extracts

A study investigated the mechanism and target of matrine in ovarian cancer via NP, MR, and molecular docking techniques. Through database screening and gene expression analysis, six core targets were identified, including TP53, CCND1, STAT3, VEGFA, IL1B, and CCL2. MR analysis indicated that TP53 and CCND1 were risk factors for ovarian cancer, whereas VEGFA and IL1B exhibited protective effects. Molecular docking confirmed the stable binding of matrine to TP53, CCND1, and IL1B. *In vitro* experiments further demonstrated that matrine downregulated CCND1 and IL1B expression while upregulating TP53, indicating that matrine possessed significant anticancer effects. These findings provide theoretical support for the potential application of matrine in oral health. The downregulation of IL1B may alleviate oral inflammation, such as periodontitis, while TP53-related mechanisms could inhibit the proliferation of oral pathogens. Additionally, CCND1 regulation may influence the oral microbiome. The antitumor mechanisms of matrine also suggest its potential in treating oral cancers and other pathological conditions (Chen and Song, 2024).

## 5 Conclusion

This review summarizes the advancements in the application of CADD technologies in drug development for oral diseases. CADD significantly enhances drug screening efficiency through molecular docking, VS, and MD simulations. However, the reliability of potential binding molecules identified solely through CADD remains uncertain. As discussed earlier, despite the identification of numerous high-affinity molecules via computational screening, only a small fraction has been proven effective upon experimental validation. For example, in the development of small-molecule drugs *that target S. mutans*, although dozens of high-binding-energy candidate molecules have been identified, only a few have exhibited efficacy in experimental validation. This underscores the necessity of experimental confirmation for CADD-derived results. Integrating wet-lab experiments, such as drug sensitivity assays, cellular studies, and animal model validations, is essential to improve the practical utility of CADD-screened molecules. Experimental validation is crucial for assessing the bioactivity and toxicity of identified molecules.

Future progress hinges on bidirectional loops between computation and experiment: standardized assay panels for key oral pathogens and inflammation/cancer models, transparent reporting of negative results to sharpen predictors, and benchmarking datasets tailored to peptide conformational diversity. Such practices will narrow the CADD–AIDD prediction–validation gap and accelerate translation in oral indications.

Furthermore, experimental feedback can be utilized to refine CADD models, thereby improving their predictive accuracy. For example, recent research on AMPs has successfully elucidated their antibacterial mechanisms by combining molecular docking with experimental validation. Similarly, in small-molecule drug development, HTVS coupled with MD simulations has significantly increased the efficiency of candidate selection. Despite existing challenges, CADD has vast potential in drug discovery for oral diseases. From AMPs to small-molecule compounds and AIDD, CADD is facilitating the transition from traditional experience-based drug discovery to a computationally driven paradigm, paving the way for precision therapies in oral diseases.
